# Global poverty estimation using private and public sector big data sources

**DOI:** 10.1038/s41598-023-49564-6

**Published:** 2024-02-07

**Authors:** Robert Marty, Alice Duhaut

**Affiliations:** World Bank, Washington, USA

**Keywords:** Environmental economics, Computer science, Statistics

## Abstract

Household surveys give a precise estimate of poverty; however, surveys are costly and are fielded infrequently. We demonstrate the importance of jointly using multiple public and private sector data sources to estimate levels and changes in wealth for a large set of countries. We train models using 63,854 survey cluster locations across 59 countries, relying on data from satellites, Facebook Marketing information, and OpenStreetMaps. The model generalizes previous approaches to a wide set of countries. On average, across countries, the model explains 55% (min = 14%; max = 85%) of the variation in levels of wealth at the survey cluster level and 59% (min = 0%; max = 93%) of the variation at the district level, and the model explains 4% (min = 0%; max = 17%) and 6% (min = 0%; max = 26%) of the variation of changes in wealth at the cluster and district levels. Models perform best in lower-income countries and in countries with higher variance in wealth. Features from nighttime lights, OpenStreetMaps, and land cover data are most important in explaining levels of wealth, and features from nighttime lights are most important in explaining changes in wealth.

## Introduction

Accurate poverty estimates are important for the design, delivery, and evaluation of social programs. However, obtaining accurate poverty estimates through household surveys is expensive and time-consuming. Many countries conduct just a couple of nationally representative surveys with poverty estimates within the span of a decade, resulting in countries relying on outdated estimates^1^. For example, among low and lower-income countries in the World Bank poverty calculator database, the latest census is 5.5 years old, and the latest nationally representative household survey is 4.5 years old^2^. Moreover, few survey clusters are surveyed multiple times, making it difficult to track changes in wealth or poverty over time at a granular level^3^.

To generate low-cost, timely, and accurate poverty estimates, a growing literature has leveraged the emerging availability of global, spatially-referenced datasets to estimate poverty^4,5^. Socioeconomic surveys are used to train machine learning models that rely on features derived from these data sources so that poverty estimates can be extrapolated across time and space. Much of the work has focused on the use of nighttime and daytime satellite imagery^1,3^; nighttime lights are a widely used proxy for local economic activity^6^, and daytime imagery can capture features such as vegetation, built-up areas, and roads—and the spatial configuration of these features. Additional research has shown the value of other data sources for poverty estimation, including anonymized cell phone data records (CDR); global human settlement data; Facebook marketing data (for example, capturing the percent of Facebook users with an expensive phone); OpenStreetMap data to understand accessibility to services like schools and health centers; and satellite-derived features beyond nighttime and daytime imagery including climate and environmental features, land use, and physical attributes such as elevation^7–14^.

With the increasing availability of global, spatially-referenced data sources, it is useful to test the value added of bringing multiple datasets together and the contexts under which poverty estimation works best. Our paper estimates an asset-based wealth index across 59 countries—spanning Africa, Asia, the Americas, and Europe—training models on data sources including daytime and nighttime satellite imagery; Facebook marketing data; weather and climate indicators; and roads and points of interest data, among others. We use these data to answer the following two related research questions. First, to what extent can multiple data sources be used to estimate levels and changes in economic status, and what is the individual contribution of each data source? Second, what are the characteristics of countries where models—including models trained on specific data sources—perform best?

Regarding the first research question, our work adds to existing research by leveraging alternative data sources to estimate wealth. For example, Pokhriyal and Jacques^7^ bring together a variety of data sources for wealth estimation—including CDR data, climate and environmental variables, and OpenStreetMap data, but focus on one country (Senegal). Chi et al.^15^ rely on data across 56 countries using features from satellite imagery, Facebook connectivity data, and OpenStreet Map, and show that mobile connectivity data derived from Facebook is among the most predictive features of wealth. Similar to^15^, we test the value added of data sources for estimating wealth across a large set of countries; while many of the data sources we test overlap with^15^, we also test additional data sources such as Facebook marketing data.

Our paper particularly fills a gap in addressing the second research question—examining the contexts under which models trained using specific data sources perform best. Some research has addressed this question, but has focused on only a limited set of countries or data sources. For example, comparing results from the Philippines and India, Fatehkia et al.^8^ show that Facebook marketing data performs similarly to satellite imagery for poverty estimation in the Philippines, but performs worse in India, where Facebook penetration is lower. Using models trained on satellite imagery across Africa, Yeh et al.^3^ shows that model performance is not significantly associated with many country-level indicators—such as country GDP, population, and urban population—but performance is lower in countries where the within-village variance in wealth is higher. Our paper extends the analysis conducted by^3^, but uses a larger and more diverse set of countries, which allows us to better test whether model performance varies by factors such as income status and geographic region. In addition, we examine the contexts under which models trained on specific data sources perform best. Consequently, we can more systematically answer questions posed by^8^ that ask whether models trained on Facebook marketing data perform best in countries with a higher share of Facebook users.

To answer these questions, we implement machine learning algorithms to estimate levels and changes of an asset wealth index across all countries with available Demographic and Health Survey (DHS) data, which spans Africa, Asia, the Americas, and Europe. DHS surveys are designed to be nationally representative and, as such, contain households across countries and across all levels of the income distribution. The asset wealth index methodology is typically used when neither income nor expenditures data are available, as is the case with DHS data. As noted by^16^, monetary measures of poverty expenditure and/or consumption data are more accurate but also more costly to collect, such that collecting simple data to build a proxy indicator of economic status might sometimes be needed. Widely used in previous machine learning and poverty-related literature, the asset wealth index has the advantage of being readily available for a larger set of countries. The index should be understood more as an indicator of economic status than a strict poverty index. It is especially useful for practical purposes, such as filling gaps between surveys or building a sampling frame when limited information on households is available, for example, to stratify surveys across the wealth distribution. Appendix S14 shows how the asset index compares with a monetary measure of poverty based on consumption/expenditure surveys produced by the World Bank to establish poverty numbers.

When estimating levels of wealth, we rely on the most recent survey from 59 countries—spanning 63,854 survey clusters (survey clusters can be interpreted as villages or neighborhoods). In estimating changes in wealth, we rely on 33 countries that have at least two DHS survey rounds (we use the latest survey and the survey closest to 2000). DHS is not designed as a panel and different villages can be surveyed across different years. Following^3^, we match clusters in one year to the nearest cluster in the survey in the previous year, only keeping pairs within 10 km of each other. Keeping only nearby pairs aims to increase the likelihood that differences in wealth between pairs over time are primarily due to changes in wealth. However, because the dataset is not a true panel, differences in wealth could still be influenced by cross-sectional spatial variation in wealth. This process creates a synthetic panel of 7714 paired clusters. To train models, we rely on globally and freely available data to ensure that our approach can be easily replicated across countries. Models are trained on an asset-based wealth index. Following^3^, we create a globally comparable index by taking the first principle component of asset and household features when pooling all countries together (see Appendix S1 for summary statistics of the global wealth index across countries). The asset-based wealth index provided by DHS is not comparable across countries. Our index is not only globally comparable, but it also has a high within-country correlation with the DHS-provided index across countries (see Appendix S2).

We train models on datasets that are (1) globally available, (2) spatially referenced, and (3) freely and publicly available. First, following much of the literature, we rely on daytime and nighttime satellite imagery. We use the average and standard deviation of values of nighttime lights and daytime imagery, and—following^1^—use features from a transfer learning approach that uses a convolutional neural network (CNN) to train daytime imagery to predict nighttime lights. CNNs rely on the values and spatial configuration of values and can detect features such as roads and the spatial configuration of roads^17,18^. To take advantage of the multi-spectral nature of daytime imagery, we estimate three separate CNN models that use (1) red, green, and blue bands as inputs; (2) NDVI (a measure of vegetation) as an input; and (3) a built-up index as input. Second, we leverage another approach to extract features from daytime imagery using the MOSAIKS (Multi-task Observation using SAtellite Imagery & Kitchen Sinks) platform^19^. MOSAIKS generates random convolutional features based on high-resolution satellite imagery from Planet. The MOSAIKS API enables extraction of these features for any location^20^. These features have been used to estimate different outcomes, including nighttime lights, income, and population density. Third, we rely on synthetic aperture radar (SAR) data from Sentinel-1. SAR data is based on transmitting waves and measuring the strength and orientation of waves reflected back to the satellite sensor^21^. Different objects scatter waves differently, and research has used SAR data for uses such as vehicle detection, crop classification, and urban change monitoring^22–24^. Fourth, we rely on Facebook advertising data that measures the proportion of the population on Facebook and the proportion of Facebook users across 34 different attributes (for example, the proportion that access Facebook using an expensive phone or the proportion that have an identified interest in luxury goods). Fifth, we use OpenStreetMap data to include the distance to and density of (1) points of interest (e.g., schools, health facilities, and restaurants) and (2) roads. Sixth, we use land cover data, including the proportion of land classified into 36 different land cover classes (e.g., built-up and cropland) from the European Space Agency Climate Change Initiative (ESA CCI) Land Cover dataset, and—following^7^—elevation and slope data. Seventh, as climate and weather contribute to food scarcity and agricultural production—particularly in rural areas—we rely on long-term climate features as well as annual temperature and precipitation^25^. Eighth, we rely on satellite-based measures of pollution. Pollution is included for two reasons. First, in some settings, poorer areas tend to be associated with higher levels of air pollution^26^. Second, pollution is an alternative source to nighttime lights to capture the extent of human activity^27^.

Table 1 summarizes the data sources and features used to train the model. Not all data sources are sufficiently available across time. Consequently, we do not use data sources such as Facebook Marketing or OpenStreetMap data when training models that estimate changes in wealth. In some cases, we rely on similar but improved data sources for estimating levels of wealth; in particular, we rely on Sentinel-2 daytime imagery and VIIRS nighttime imagery to train CNN models when estimating levels of wealth, but rely on Landsat and DMSP-OLS to train CNN models when estimating changes in wealth.

We implement a supervised machine learning model to estimate the globally-comparable wealth index. As we use a large number of features, we train models using algorithms that can handle high-dimensional data; when estimating levels of wealth, we rely on 520 features across the different data sources. We test training models using the Extreme Gradient Boosting (XGBoost) algorithm, regularized linear regression (lasso, ridge, and elastic net), and support vector machines^28–30^. We train models using all features and using only specific sets of features—such as models only trained on data from OpenStreetMap data, Facebook data, etc.

We use four different approaches for developing training and test sets: (1) within-country models, training the model only using a sub-sample of countrywide data and testing the performance of the model using the remainder; (2) within-continent estimation, where—for each country—we train a model using all other countries in the continent to estimate wealth in the country; (3) other-continent estimation, where we train a model using each pair of continents and estimate wealth in the other continent; and (4) global estimation, where we train a model on all other countries to estimate wealth in a country. The approaches allow for testing the trade-off between using less training data in a similar context and more data in less similar contexts.

**Table 1:** Summary of data sources for wealth estimation.

Source	Time span	Level/change	Features for wealth estimation
Daytime and Nighttime Satellite Imagery			
VIIRS	2012–Present	Level	Nighttime lights: Average, standard deviation over time, and standard deviation over space
Harmonized DMSP-OLS and VIIRS	1992–2021	Changes	Nighttime Lights: Average and standard deviation over space
Landsat 7	1999–2021	Both	Spectral bands and indices (NDVI and build-up index): Average, standard deviation over time, and standard deviation over space
–	–	Changes	Convolutional neural network used to train daytime imagery on nighttime lights; features extracted from CNN
Sentinel-2	2015–Present	Levels	Convolutional neural network used to train daytime imagery on nighttime lights; features extracted from CNN
MOSAIKS	2019	Levels	Features extracted from high-resolution daytime imagery
Synthetic Aperture Radar Data			
Sentinel-1	2014–Present	Levels	Synthetic aperture radar data, measuring the average and standard deviation of VV and VH signals, and the ratio of the two—VV/VH. VV indicates vertical transmit, vertical receive, and VH indicates vertical transmit, horizontal receive
Facebook marketing data			
Facebook	Present	Levels	Proportion of monthly active Facebook users according to select attributes (e.g., proportion of Facebook users with an iPhone)
Roads and points of interest			
OpenStreetMap	Present	Levels	(1) Number of points of interest (POIs) near survey (all and by type—e.g., restaurants, schools, health facilities, etc), (2) distance to nearest POI (all and by type), (3) length of roads near survey (all and by type—e.g., trunk roads and primary roads), and (4) distance to nearest road (all and by type)
Land Cover and Type			
European Space Agency Climate Change Initiative (ESA CCI) Land Cover	1992–2018	Both	Proportion of area near survey classified according to 36 different land cover classes
Shuttle Radar Topography Mission (SRTM)	Time-Invariant	Levels	Average elevation and slope
Weather and Climate			
WorldClim	Average of 1970–2000	Levels	19 bioclimatic variables, including annual mean temperature, annual precipitation, and mean temperature of the wettest quarter
European Centre for Medium-Range Weather Forecasts: ERA5	1979–2020	Both	Average annual precipitation and temperature
Pollution			
Sentinel-5P	2018–Present	Levels	Average pollution levels from six metrics: nitrogen dioxide, carbon monoxide, sulfur dioxide, ozone, formaldehyde, and an aerosol index
MODIS	2000–Present	Both	Aerosol optical depth

## Results

### Correlation of features to asset wealth

**Figure 1:**
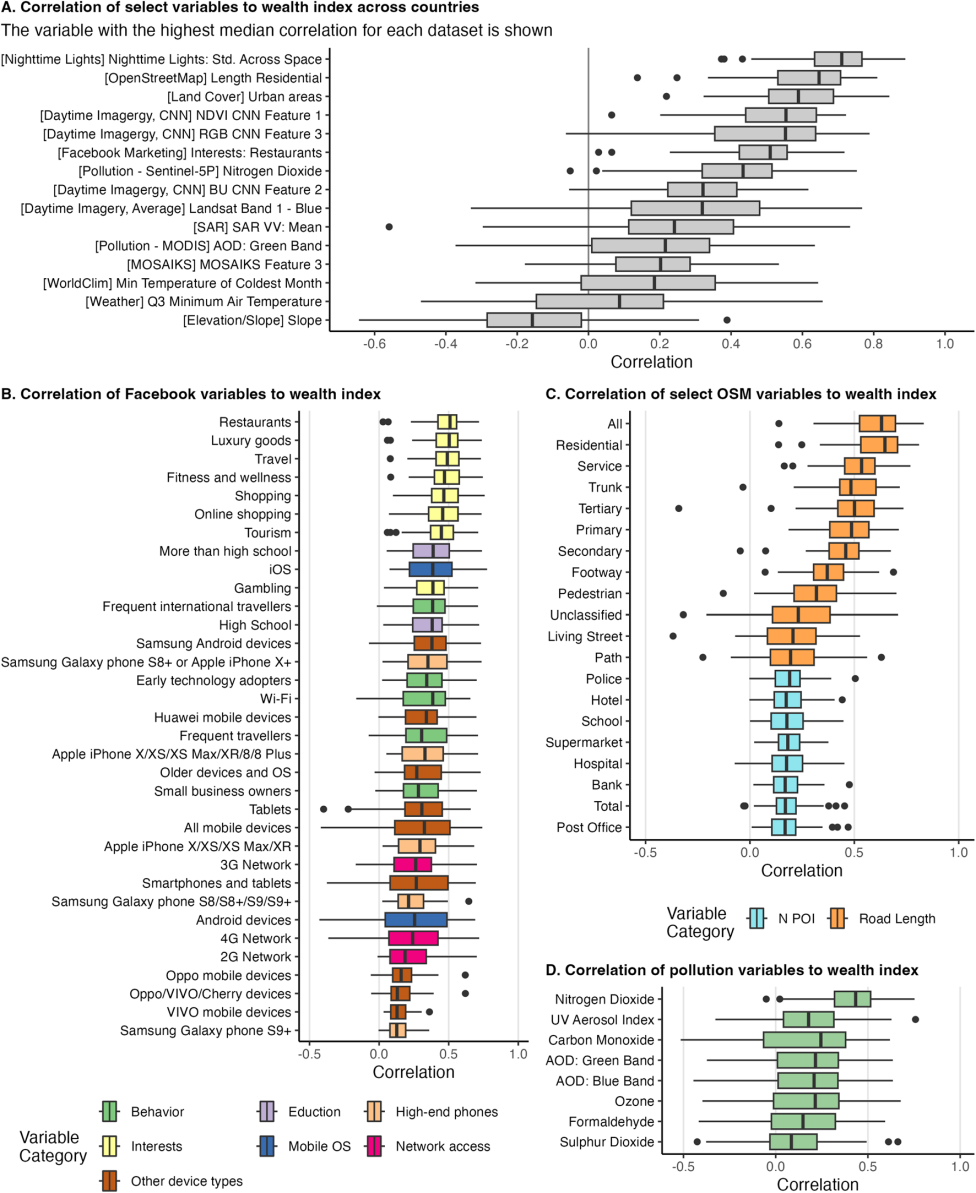
Distribution of within-country correlations of select variables to levels of wealth. Correlations are computed at the cluster level using the latest survey year for each country. **Panel A** shows the distribution of within-country correlations of the feature with the highest median correlation across countries for each dataset. **Panel B** shows the correlation of all variables from the Facebook marketing data. **Panel C** shows select variables from OpenStreetMap data; the panel shows all variables of (1) the length of different classes of roads and (2) the number of different points of interest (POIs) near survey clusters. **Panel D** shows the correlation of all pollution variables from Sentinel-5P and MODIS; AOD variables are from MODIS and the other variables are from Sentinel-5P. The boxplots include center line, median; box limits, upper and lower quartiles; whiskers, 1.5$\times$ interquartile range; points beyond whiskers, outliers.

Before turning to the results from the machine learning models, we first examine the correlation of features to levels and changes in wealth. Figure 1 shows the correlation of features to levels of wealth; panel A shows the distribution of within-country correlations for the feature with the highest median correlation in each dataset. Overall, individual features across most datasets have a relatively strong correlation with wealth; features from nighttime lights, OpenStreetMaps, land cover, CNN models, and Facebook marketing data all see median correlations near or above 0.5. Panels B–D show distributions of within-county correlations across features from Facebook, OpenStreetMaps, and pollution datasets. Across all features from Facebook marketing data (panel B), the 25th percentile correlation is greater than zero; the proportion of Facebook users with interests in restaurants, luxury goods, and travel have the highest median correlations of approximately 0.5 (Appendix S3 shows the correlation of all Facebook features to the wealth index for each country). Panel C shows that—among features from OpenStreetMaps—the length of all roads and residential roads have high correlations with wealth; the 25th percentile of within-country correlations is above 0.5 for both variables. Among pollution variables, nitrogen dioxide is unique in having a large, positive correlation with wealth for most countries; other pollution variables show a much smaller correlation (while Fig. 1 shows the correlation of top features to wealth, see Appendix S4 which shows the correlation between top features).

**Figure 2:**
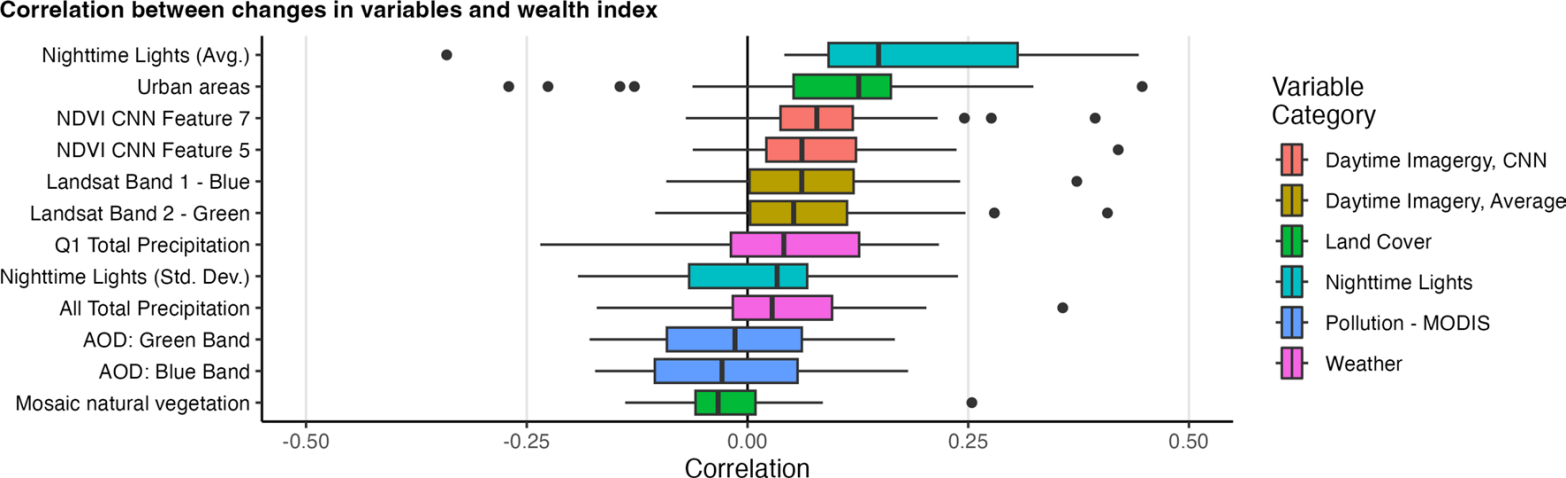
Distribution of within-country correlation of changes in select variables to changes in wealth, using clusters as the unit of analysis. We show the two variables with the highest median correlation for each dataset. The boxplots include center line, median; box limits, upper and lower quartiles; whiskers, 1.5$\times$ interquartile range; points beyond whiskers, outliers.

Figure 2 shows the correlation of changes in features to changes in wealth; the figure shows the distribution of within-country correlations for the top two features with the highest median correlation in each dataset. Overall, correlations of changes are lower than that of correlations of levels. However, changes in nighttime lights, urban areas, and select features from CNNs generally see positive correlations for most countries, with some countries seeing relatively high correlation coefficients. For example, the average correlation between changes in nighttime lights and the wealth index is 0.18, with a maximum correlation of 0.44 (in Mozambique).

### Machine learning model performance

#### Estimating levels of wealth

**Figure 3:**
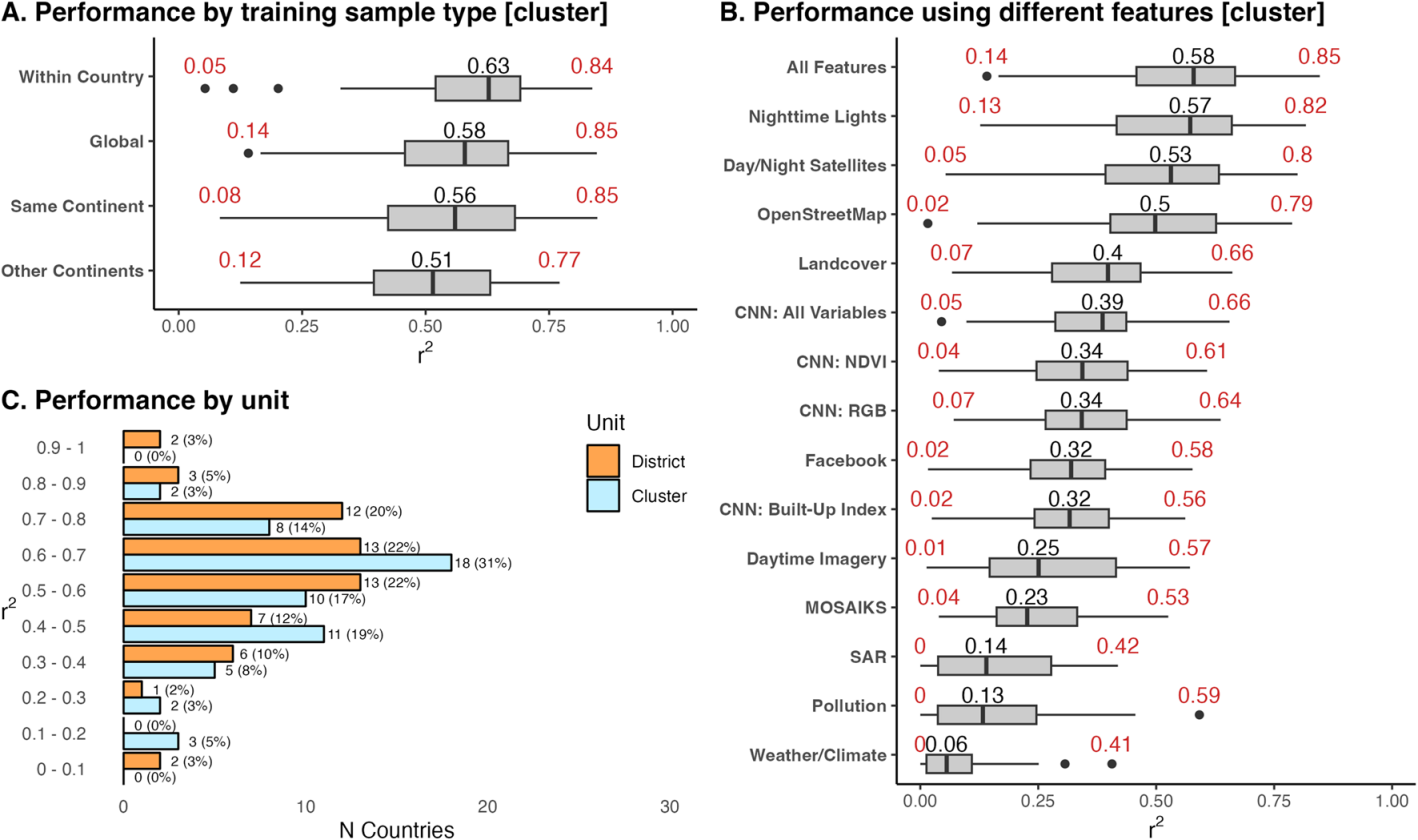
Distribution of model performance across countries, explaining levels of wealth. The black number shows the median and the red numbers show the minimum and maximum $r^2$. **Panel A** shows model performance by the sample used to train countries. **Panel B** shows model performance when using different sets of features to train models. **Panel C** shows the distribution of model performance at the village and district level. **Panels B,C** show results where models for each country are trained on data from all other countries (the global training sample approach). The boxplots include center line, median; box limits, upper and lower quartiles; whiskers, 1.5$\times$ interquartile range; points beyond whiskers, outliers.

Models trained on only data within the country perform best, indicating that models work best when trained on more similar—albeit less—data (Fig. 3, panel A). Training on only within-country data performs best for 58% (N = 34) countries, followed by training on countries in the same continent (20%; N = 12), followed by training on all other countries globally (19%; N = 11), followed by training on countries in other continents (3%; N = 2). Countries where within-country training performed best tend to have more clusters (median = 520 clusters; 75th percentile = 807) than countries where training on other countries performed best (median = 341 clusters; 75th percentile = 524). This finding indicates that training on data from other countries is particularly advantageous for countries with less survey data to train models on.

**Figure 4:**
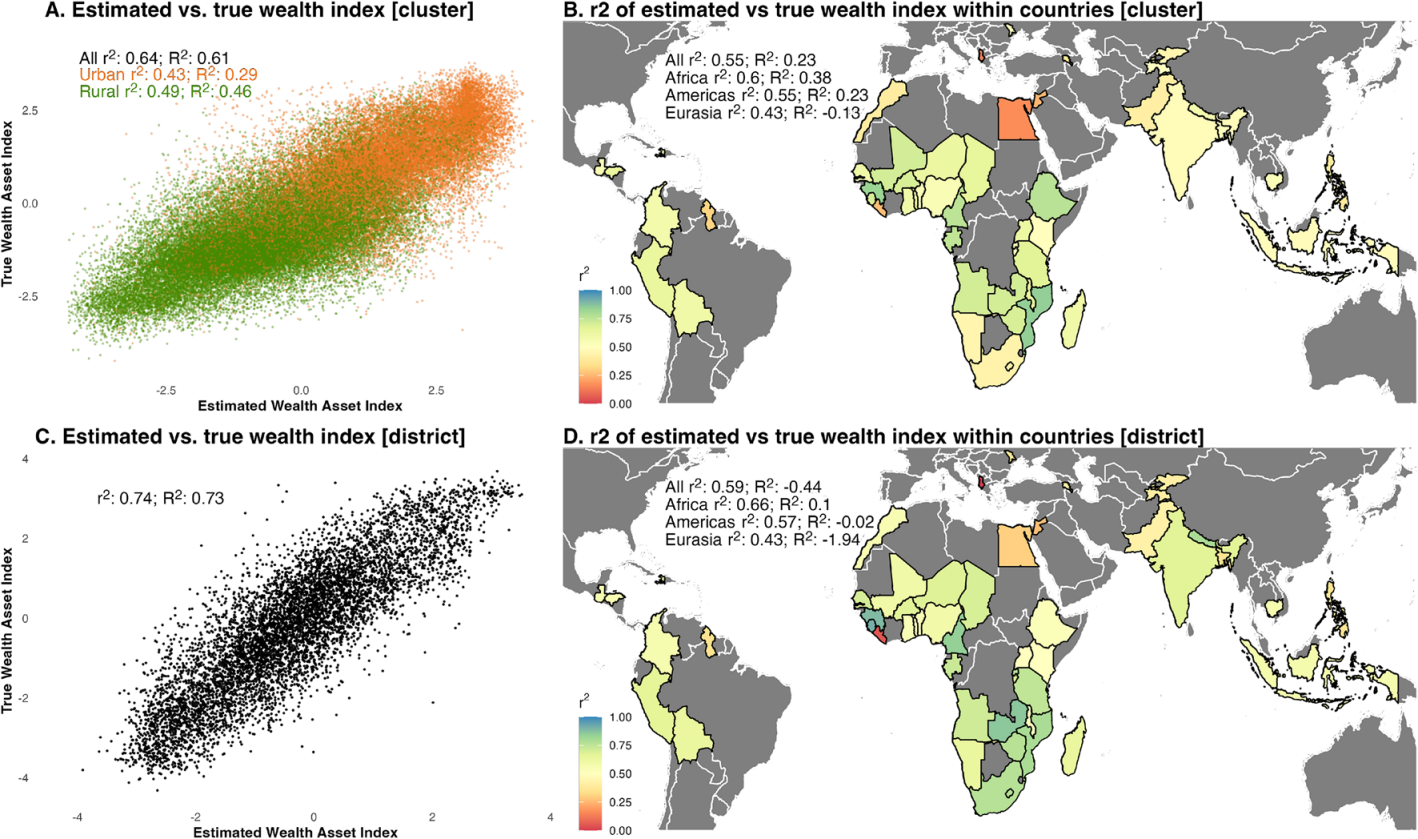
Performance of models estimating levels of wealth index. **Panel A** shows a scatterplot of the estimated and true wealth indices. **Panel B** shows model performance when considering individual countries. **Panels C**,**D** are similar to panels A and B, but using results aggregated at the district level. All panels use results where models for each country are trained on data from all other countries (the global training sample approach). $r^2$ is the squared Pearson correlation coefficient, and $R^2$ is the coefficient of determination. The maps in panels B and D were produced using R, version 4.2.2 (https://www.r-project.org/); data to produce the country-level basemaps come from Natural Earth (https://www.naturalearthdata.com/).

Relying on models trained on all other countries and pooling data across countries, our model explains 64% of the variation in wealth at the cluster level and 74% of the variation when aggregating data to the district (second administrative division) level (Fig. 4, panels A,C; also see Fig. 3, panel C for comparison of cluster and district results; Appendix S5 shows results for each country, and Appendix S6 shows pooled results for each continent). To facilitate comparing our results with similar studies^1,3^, we rely on the squared Pearson correlation coefficient ($r^2$) as our main measure of performance; however, we also report the coefficient of determination ($R^2$)—calculated as $R^2 = 1 - \frac{\sum (y_i - \hat{y}_i)^2}{\sum (y_i - \bar{y})^2}$. $R^2$ measures how well the true and estimation values match. In many cases, such as Mozambique, the $r^2$ and $R^2$ are similar, indicating that the true and estimated values both move together and are similar; however, in other contexts, the $R^2$ is notably lower than the $r^2$. For example, in Sierra Leone, estimated and true wealth move strongly together ($r^2$ = 0.7), but estimated wealth is systematically larger than true wealth ($R^2$ = 0.21; see Appendix S5).

For many countries, aggregating to the district level notably improves model performance; for example, for India, the $r^2$ increases from 0.48 at the cluster level to 0.67 at the district level. Better performance at higher levels of aggregation is also seen in other papers, such as^3^. We find that across most countries, there is a larger variation in wealth across districts than within districts (see Appendix S12); averaging cluster-level results within districts with relatively similar wealth levels may help to average out error from individual clusters, but effectively capture the overall wealth of the district.

Separating across urban and rural clusters, the model explains 43% of the variation in wealth in urban areas and 49% of the variation in rural areas. The model performs best in Africa ($r^2$ = 0.6, on average across countries at the cluster level, and $r^2$ = 0.66 at the district level), followed by the Americas ($r^2$ = 0.55 at the cluster level; $r^2$ = 0.57 at the district level) and Eurasia ($r^2$ = 0.43 at the cluster and district levels) (Fig. 4, panels B and D; Appendix S5 shows scatterplots of true and estimated wealth for each country).

Models trained using all features see the best results, where the median $r^2$ across countries is 0.58, and the minimum $r^2$ is 0.14 (Fig. 3, panel B). Models using only features from specific data sources—such as OpenStreetMaps or nighttime lights—only perform slightly worse, with a median $r^2$ of 0.57; however, some countries see an $r^2$ near zero when training on individual data sources. While training models on only specific sets of features may work well for some countries, training models on features from multiple datasets helps to ensure good model performance across all countries (Appendix S7 shows model performance for each country using each set of features).

Appendix S14 shows a sensitivity analysis when training models to estimate a similar asset-based wealth index and a consumption indicator, using data from six countries from the Living Standards Measurement Survey (LSMS). We leverage LSMS data for this comparison as DHS does not provide consumption or expenditure data, while LSMS provides data on both consumption and assets. The asset and consumption indicators are significantly associated, where the asset index explains up to 66% of the variation in consumption. Machine learning models work better to estimate the wealth index compared to consumption, and similar sets of features are most important in estimating both the wealth index and consumption.

**Figure 5:**
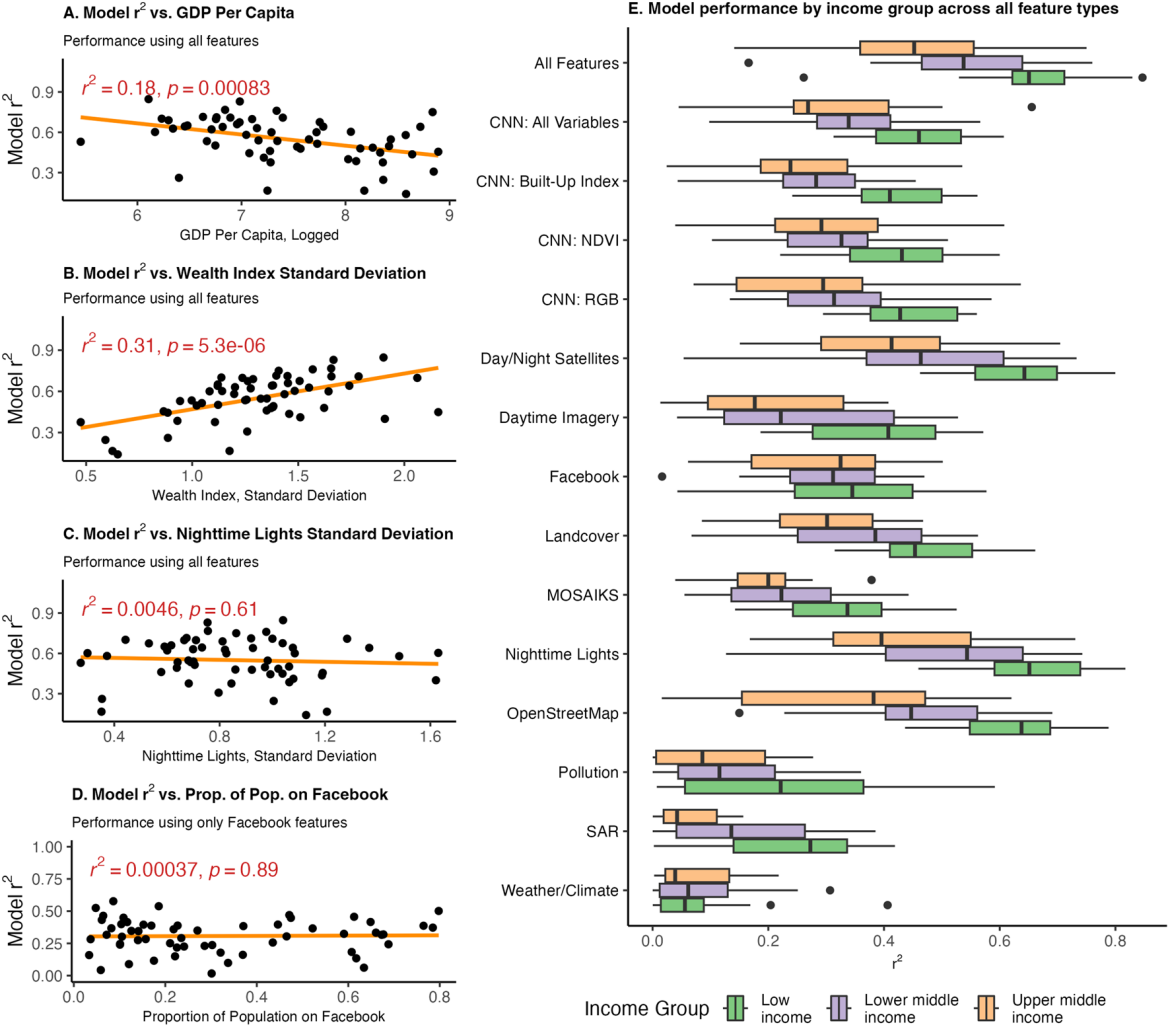
Determinants of the variation in model performance across countries, explaining levels of wealth. **Panel A** shows the association between GDP per capita and model performance. **Panel B** shows the association between the wealth index standard deviation and model performance. **Panel C** shows the association between the nighttime lights standard deviation and model performance. **Panel D** shows the association between the proportion of the population on Facebook—measured using monthly active users divided by a country’s population—and model performance using only Facebook features to train the model. **Panel E** shows the distribution of model performance by income level using models trained across different feature sets. The boxplots include center line, median; box limits, upper and lower quartiles; whiskers, 1.5$\times$ interquartile range; points beyond whiskers, outliers. All panels use results where models for each country are trained on data from all other countries (the global training sample approach), and where the unit of analysis is clusters.

Figure 5 uses a number of country-level variables to explain where the models best explain wealth. Models work best in countries with more variation in the wealth index and lower GDP per capita (Fig. 5, panels A,B). Models trained using only Facebook marketing features perform similarly across countries with low and high proportions of the population on Facebook (Fig. 5, panel D). Models perform best in lower-income countries across all feature types, with the exception of Facebook variables, pollution, and weather/climate variables—where models generally perform similarly across all income levels (Fig. 5, panel E). Appendix S9 explains variation in wealth estimate errors at the cluster level, showing that clusters with the highest nighttime lights tend to have the least error; in addition, error estimates tend to be slightly lower in rural locations, in Africa, and in low and lower middle-income countries compared to upper middle-income countries.

Appendix S13 compares model performance to results from similar papers. When averaging model performance across countries, our results are slightly worse than those of^15^ ($r^2$ of 0.55 vs. 0.59, considering all countries with DHS data) and to those of^3^ ($r^2$ of 0.70 vs. 0.6, considering all African countries with DHS data). However, when pooling results across countries, our results estimating levels of wealth at the cluster level improve on those of^3^: $r^2$ of 0.75 vs. 0.67 for all African countries; $r^2$ of 0.61 vs. 0.32 when just considering urban clusters; and $r^2$ of 0.65 vs. 0.32 when just considering rural clusters^3^.

#### Estimating changes in wealth

**Figure 6:**
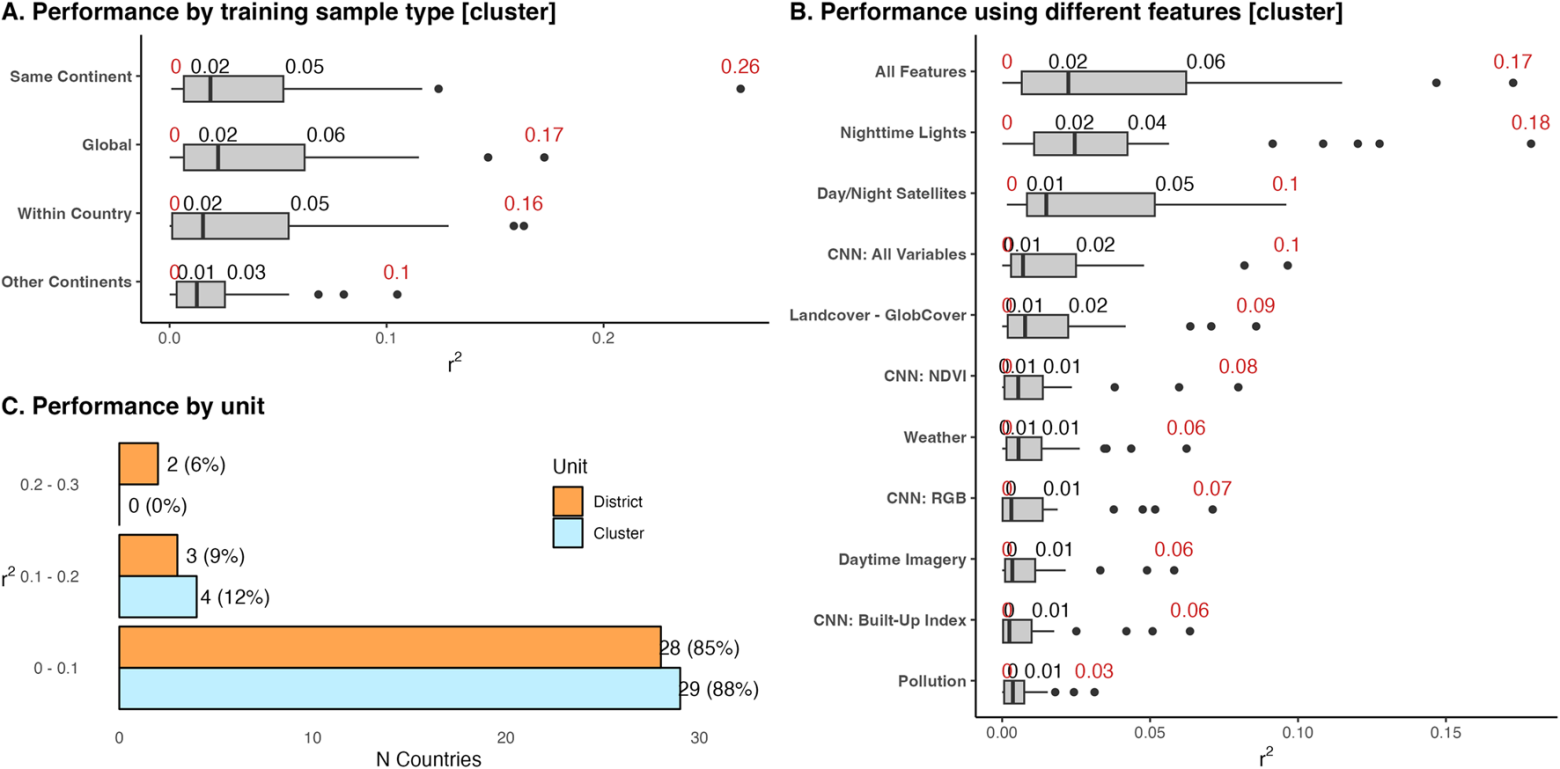
Distribution of model performance across countries, explaining changes in wealth. The black number shows the median and the red numbers show the minimum and maximum $r^2$. **Panel A** shows model performance by the sample used to train countries. **Panel B** shows model performance when using different sets of features to train models. **Panel C** shows the distribution of model performance at the village and district level. **Panels B,C** use results where models for each country are trained on data from all other countries (the global training sample approach). The boxplots include center line, median; box limits, upper and lower quartiles; whiskers, 1.5$\times$ interquartile range; points beyond whiskers, outliers.

The median model performance ($r^2$) is 2%; at the cluster level and 4% at the district level (see Fig. 6; Appendix S8 shows results for each country). While these values are low, there is notable variation in model performance—in some countries, models explain up to 17% of the variation in changes in wealth at the cluster level and 26% at the district level. Consequently, while the models generally perform poorly, the results show that—in select cases—model estimates are informative in explaining some variation in changes of wealth. Models trained on all features perform best, followed by models trained on nighttime lights, and models trained on both day and nighttime imagery (Fig. 6, panel B).

**Figure 7:**
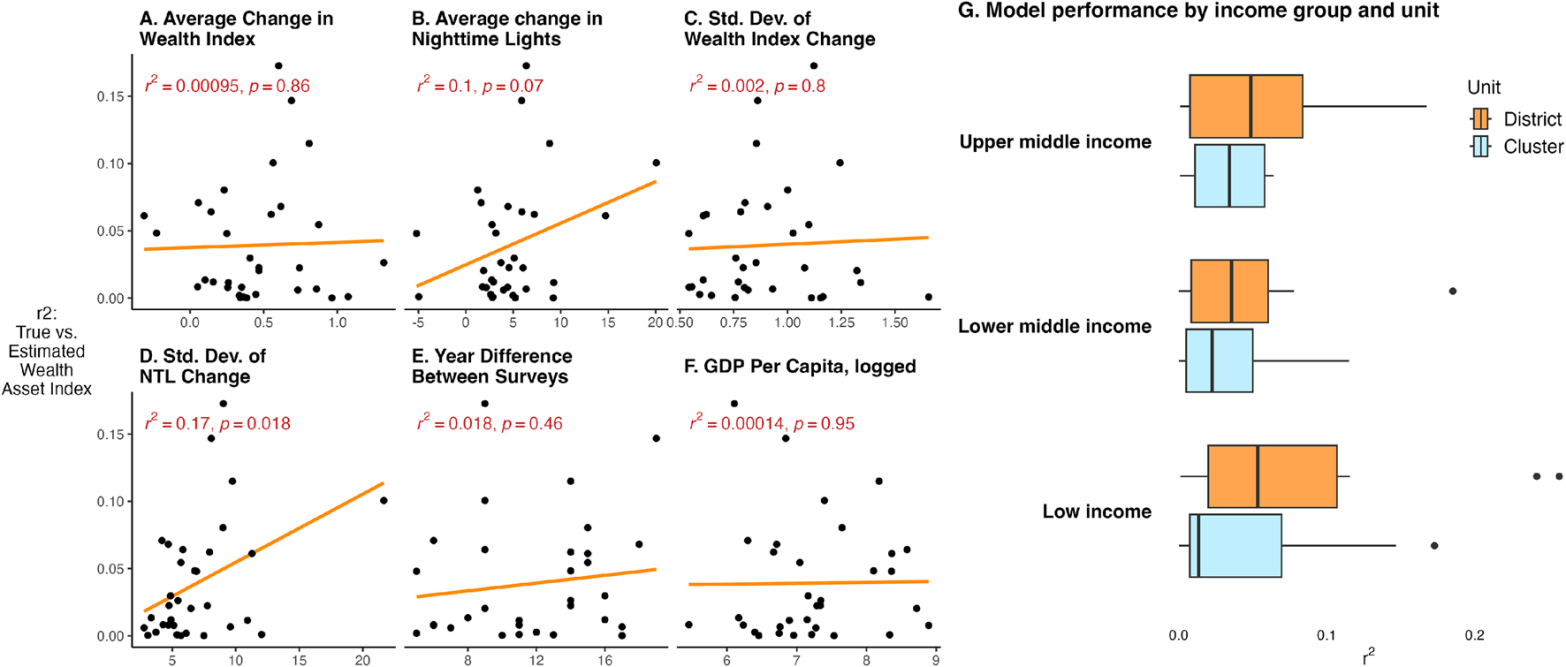
Explaining variation in model performance across countries, explaining changes in wealth. The figure shows the association between model performance and average changes in wealth (**Panel A**), average changes in nighttime lights (**Panel B**), the standard deviation of the change in wealth (**Panel C**), the standard deviation of the change in nighttime lights (**Panel D**), the years between surveys (**Panel E**), and current GDP per capita (**Panel F**). **Panel G** shows the distribution of model performance by income level, using villages and when aggregating to the district level. The boxplots include center line, median; box limits, upper and lower quartiles; whiskers, 1.5$\times$ interquartile range; points beyond whiskers, outliers.

Figure 7 shows that model performance tends to be slightly higher in countries where there is a larger change in nighttime lights (panel B) and variation in the change in nighttime lights (panel D). Other country-level variables—such as changes in the wealth index and year difference between surveys—do not explain variation in model performance. Appendix S9 explains variation in wealth estimate errors at the cluster level. Similar to models explaining levels of wealth, error estimates tend to be slightly lower in rural locations, in Africa, and in low and lower middle-income countries compared to upper middle-income countries. Appendix S13 shows that model performance explaining changes in wealth is not as high as results from^3^; Yeh et al.^3^ uses a different approach with their machine learning algorithm, including in their use of convolutional neural networks, which could explain their better performance.

## Application: estimating wealth in different years

**Figure 8:**
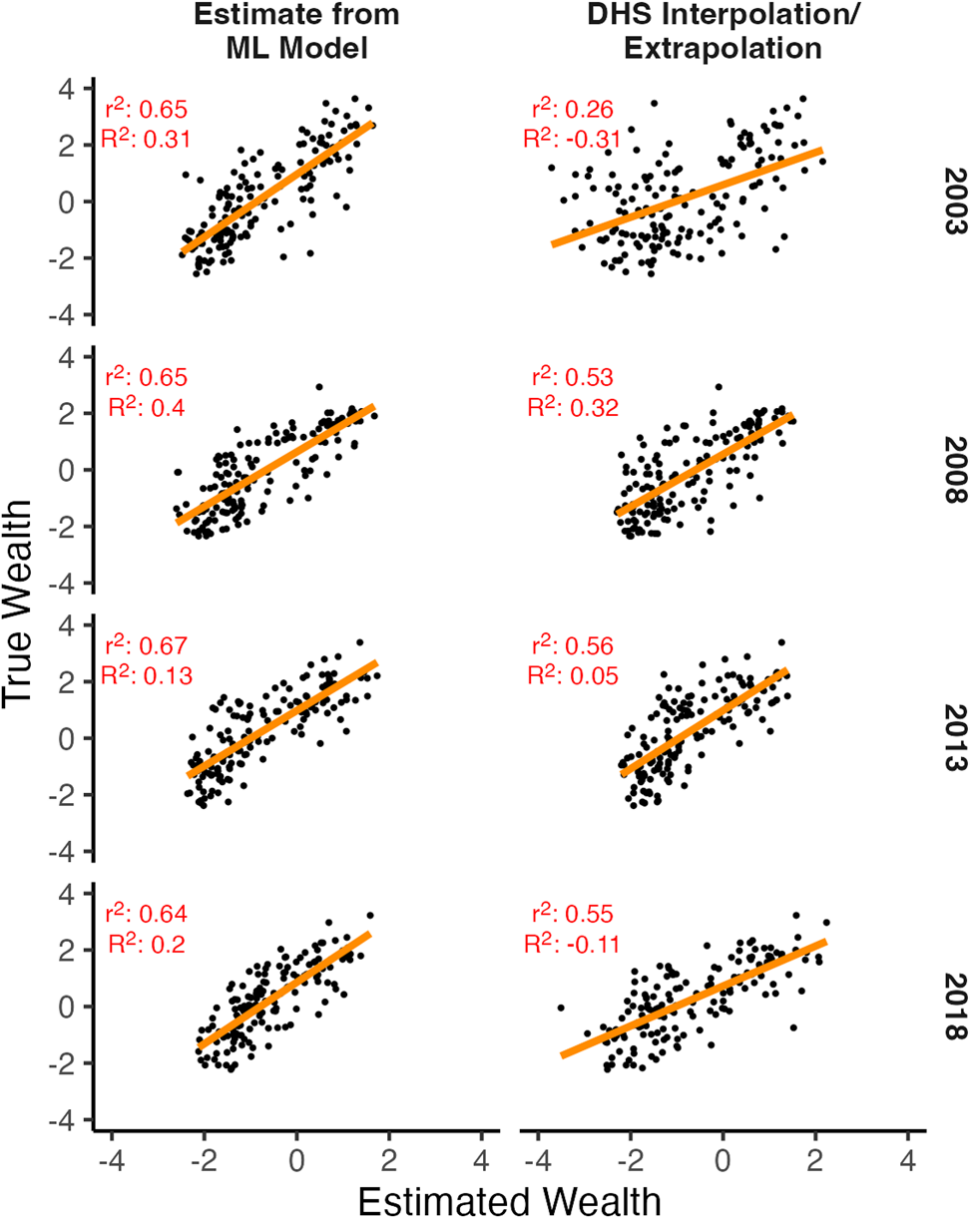
Comparison of true wealth estimates versus estimates from machine learning model and using DHS data to interpolate and extrapolate wealth estimates; data is extrapolated for 2003 and 2018 data, and interpolated for 2008 and 2013. For each year, the two closest DHS rounds are used to estimate wealth; for example, data in 2003 and 2013 is used to interpolate wealth for 2008. Wealth estimates are at the second administrative division level. The $r^2$ is the squared Pearson correlation coefficient, and $R^2$ is the coefficient of determination.

An application of this work is to estimate wealth during time periods when survey data is not available, for example, to create a robust sampling frame to evaluate access to public services or programs. An alternative method sometimes used by researchers and practitioners is to interpolate or extrapolate existing data to years where data is missing. Using a case study of estimating wealth at the second administrative level in Nigeria, we test our model performance against a survey interpolation/extrapolation approach in the most populous country in Africa. Nigeria has four rounds of DHS data: 2003, 2008, 2013, and 2018. For the machine learning approach, we train a model using features that are available across all time periods; we train data on all countries except Nigeria using each country’s latest DHS survey round. We estimate results at the cluster level, and aggregate results to the second administrative division level. We compare these estimates to interpolating and extrapolating wealth based on DHS survey rounds; data are aggregated to the second administrative level before interpolating/extrapolating. For each year, the two closest DHS rounds are used to estimate wealth; for example, data in 2003 and 2013 are used to interpolate wealth in 2008. Estimates in 2008 and 2013 are interpolated, while estimates in 2003 and 2018 are extrapolated. For each administrative region, estimates are linearly interpolated or extrapolated over time based on values from the same administrative region in different years. Figure 8 shows that the machine learning wealth estimates are more strongly associated with true wealth compared to interpolating and extrapolating wealth from DHS data. Extrapolating wealth particularly performs poorly compared to machine learning estimates. Appendix S10 shows similar results when using data aggregated to the first administrative level.

## Discussion

This paper shows that global, freely available, and spatially referenced data can estimate levels of wealth with a relatively high degree of accuracy—while estimating changes in wealth is more difficult. The results show notable variation in model performance across countries, but show that they perform the best in countries where they can be most useful: countries with more variation in wealth, and in lower-income countries. The results also demonstrate the value added of using features from a variety of different data sources. The best performing models use data from all data sources. Models trained on features from just OpenStreetMaps, nighttime lights, or land cover data also perform well—on average—but there is large variation in model performance across countries, where the model explains virtually none of the variation in wealth in some countries when trained on some individual data sources. Models trained on features from all data sources guard against poor model performance in specific countries; across countries, the models estimating levels of wealth leveraging all features explain—at minimum—14% of the variation in wealth.

Some data sources provide features that are more interpretable than others. Beyond using features to estimate wealth, interpretable features can provide a richer context of characteristics of locations—this is particularly true for OpenStreetMaps and Facebook marketing data. For example, from OpenStreetMaps, the density of residential roads near a location is strongly correlated with wealth. Examining variation in levels and changes in residential roads could be useful to understand the level of amenities provided to richer households. Features from Facebook Marketing data can similarly provide an informative understanding of populations—such as whether Facebook users are frequent travelers or whether they connect to Facebook from an expensive phone. When analyzing features individually, it is also important to consider potential biases. For example, OpenStreetMap relies on crowdsourced data such that some countries may have more complete data than others. Growth in roads could simply result from more complete data being added to OpenStreetMaps. Similarly, Facebook data could be biased as only certain segments of the population may be on Facebook (Appendix S15 explores this bias by analyzing the association between a variable captured by both DHS and Facebook Marketing data—high school completion. We find that the variables move roughly together, particularly at the district level, which indicates that specific Facebook features can capture specific attributes of populations beyond wealth in some contexts). The strong association between OpenStreetMaps and Facebook variables with wealth shows that these data sources can capture on-the-ground dynamics—but additional work could inform further ground truthing these data sources.

Overall, the paper shows the promise of leveraging new, globally available data sources for estimating wealth in contexts where it matters the most: for lower-income countries, and when there is more variation in wealth. All data sources come with their own sets of biases and limitations. However, integrating data across different sources—from satellites to social network data to crowdsourced maps data from OpenStreetMaps—can help to overcome the limitations of any individual data source.

## Methods

### Preparation of survey data and wealth index

We rely on survey data from the Demographic and Health Surveys (DHS), which has become a common data source for training models for poverty estimation due to its wide coverage. We use data from all countries with available standard DHS data with associated GPS coordinates of the survey clusters. For estimating levels of wealth, we rely on the most recent survey for each country, resulting in 63,854 survey clusters across 59 countries. For estimating changes in wealth, we rely on the most recent survey round and the oldest survey that was implemented closest to 2000, which results in a dataset capturing 7714 survey clusters across 33 countries. As the coverage of some data sources, particularly Landsat 7 and MODIS, started in 2000, we limit older surveys to those implemented near 2000. This process results in including two countries with surveys implemented in 1998, which assumes that data from sources such as Landsat 7 and MODIS captured in 2000 should still be indicative of 1998. DHS data are representative both nationally and at the first administrative division level, where DHS provides a geographic coordinate for each cluster^31^.

To protect the privacy of respondents, DHS randomly displaces the true geographic location. Coordinates of urban clusters are displaced up to 2 km, and most coordinates of rural clusters are displaced up to 5 km—with 1% displaced up to 10 km^32^. For most features, we extract values within 2.5 km of the reported survey cluster location (e.g., average and standard deviation of nighttime light values within 2.5 km of the survey cluster). This process ensures that all urban clusters appear within the location used to extract values from features. Some rural clusters may appear outside the location used to extract values from features. However, especially in rural locations, we may expect positive spatial autocorrelation for a number of features—such that features are similar in nearby areas.

From DHS, we use a set of variables that capture the relative wealth of survey clusters. DHS provides a pre-computed wealth index, which is the first principal component of socio-economic attributes at the household level (e.g., roof material or owns assets such as a television). The wealth index is computed within each country, so values from one country cannot be compared with an index from another country or within the same country over time. To remediate that issue, we follow an approach by^3^ and use a set of socio-economic variables to construct a wealth index that is comparable across countries. The index is created by using the first principle component of the variables, a standard approach to creating asset or wealth indices^33^. We use the following variables: ownership of assets including a television, fridge, motorbike, and car; access to electricity; quality of floor, wall, and roof material (each categorized on a scale from 1 to 3 as natural, rudimentary, or finished floor, roof or walls); time to get drinking water (categorized on a scale from 1 to 3 as more than 30 min, > 0–30 min, and 0 min, or drinking water in household); whether the household has a flush toilet connected to the sewer system; whether the household has water piped into their dwelling; and the number of people sleeping per bedroom (we divide the number of people listed in the household by the number of sleeping rooms and categorize this variable on a scale from 1 to 3 as more than two people per room, one to two people per room and less than one person per room).

Not all the above variables are available for the earlier survey rounds. Consequently, when estimating levels of wealth—which rely on the most recent survey year—we include all the above variables when computing the principal component. When developing a wealth index to capture changes in wealth—which relies on earlier DHS surveys—we include a more limited set of features that are available in all survey rounds. Here, we use the ones above but exclude the number of people sleeping per bedroom, wall material, roof material, and time to get drinking water. Both indices (the index created from the full set of features and the index created from the limited set of features) are highly correlated with each other (cor = 0.981). On average across countries, the index using the full set of features has a 0.917 correlation with the DHS wealth index, and the index using the limited set of features has a 0.864 correlation with the DHS wealth index.

### Preparation of data for poverty estimation

We rely on a variety of globally available and spatially referenced data sources to use as features for estimating wealth. Datasets include raw satellite imagery, data derived from satellite imagery, private sector data, and data derived from crowdsourcing. In this section we describe each of the datasets and variables extracted from the datasets; Table 1 lists the different datasets and the features extracted from each dataset.

#### Nighttime lights

Nighttime lights have been shown to be a strong proxy of local economic activity^6,34–37^ and measures of welfare, including wealth estimates derived from DHS^38^. When estimating levels of wealth, we rely on the Visible Infrared Imaging Radiometer Suite (VIIRS), which has captured nighttime lights since 2012 at roughly a 500-m resolution. We take the average value of nighttime lights within 2.5 km of each survey cluster, the standard deviation of nighttime lights over space, and the standard deviation of nighttime lights over time (using monthly nighttime lights data) using the year before, during, and after the survey year. When estimating changes in wealth, we rely on a dataset from^39^ who harmonizes nighttime lights from DMSP-OLS (available from 1992 to 2013) and VIIRS (available from 2012 to present) into a single dataset. Raw DMSP-OLS data is at a lower resolution than VIIRS, and suffers from issues such as blooming where light affects surrounding pixels^40^. The harmonized dataset uses VIIRS data to simulate DMSP-OLS-like data, so that trends in nighttime lights can be measured across DMSP-OLS and VIIRS time periods. The harmonized dataset is available annually from 1992 to 2021; we take the average and standard deviation values of nighttime lights within 2.5 km of each survey cluster.

#### CNN features from daytime imagery predicting nighttime lights

We follow previous research that use convolutional neural networks (CNNs) to extract features from satellite imagery. CNNs capture the spatial configuration of images, not just average values from imagery; consequently, CNNs may capture features such as roads, buildings, vegetation and how these are arranged (e.g., buildings in neat rows or a more disorganized layout)^17^. We follow previous studies that use a transfer learning approach, whereby a CNN model using daytime imagery as features is used to predict the magnitude of nighttime lights^1,41^. The features created from this model are then used in a separate model to estimate wealth. Given that nighttime lights is widely recognized as a strong proxy for economic activity and wealth^6^, a CNN predicting nighttime lights will develop features that are also relevant for capturing wealth.

For estimating levels of wealth, we rely on daytime imagery from Sentinel-2^42^. Sentinel-2 has a 10-m resolution; surface reflectance data has been available since 2017 and captures imagery roughly every week. For each 10-m pixel, we use the median value across images from 2017 to 2020. Using data across multiple years follows^3^, who note that using a longer time period minimizes the impact of cloud cover and minimizes distortions by seasonal or short-run variations in imagery. One disadvantage of using Sentinel data is that many DHS surveys were administered before 2017. However, previous research faces a similar issue and observes strong results, indicating that imagery from later years still provides useful information about wealth in past years. For example, Jean et al.^1^ relies on satellite imagery from Google Maps from 2013 to 2015 to estimate poverty in 2010–2013. An alternative approach would be to use Landsat data, where imagery goes back decades; however, we opt for Sentinel as it has a finer spatial resolution than Landsat’s 30-m resolution. For estimating changes in wealth, we do rely on Landsat imagery. We rely on Landsat 7, which provides imagery from 1999 to 2022 at a 30-meter resolution. Similar to how we process Sentinel-2 data, we use data from the year before, during, and after the survey year—taking the median value for each pixel across these years.

**Figure 9:**
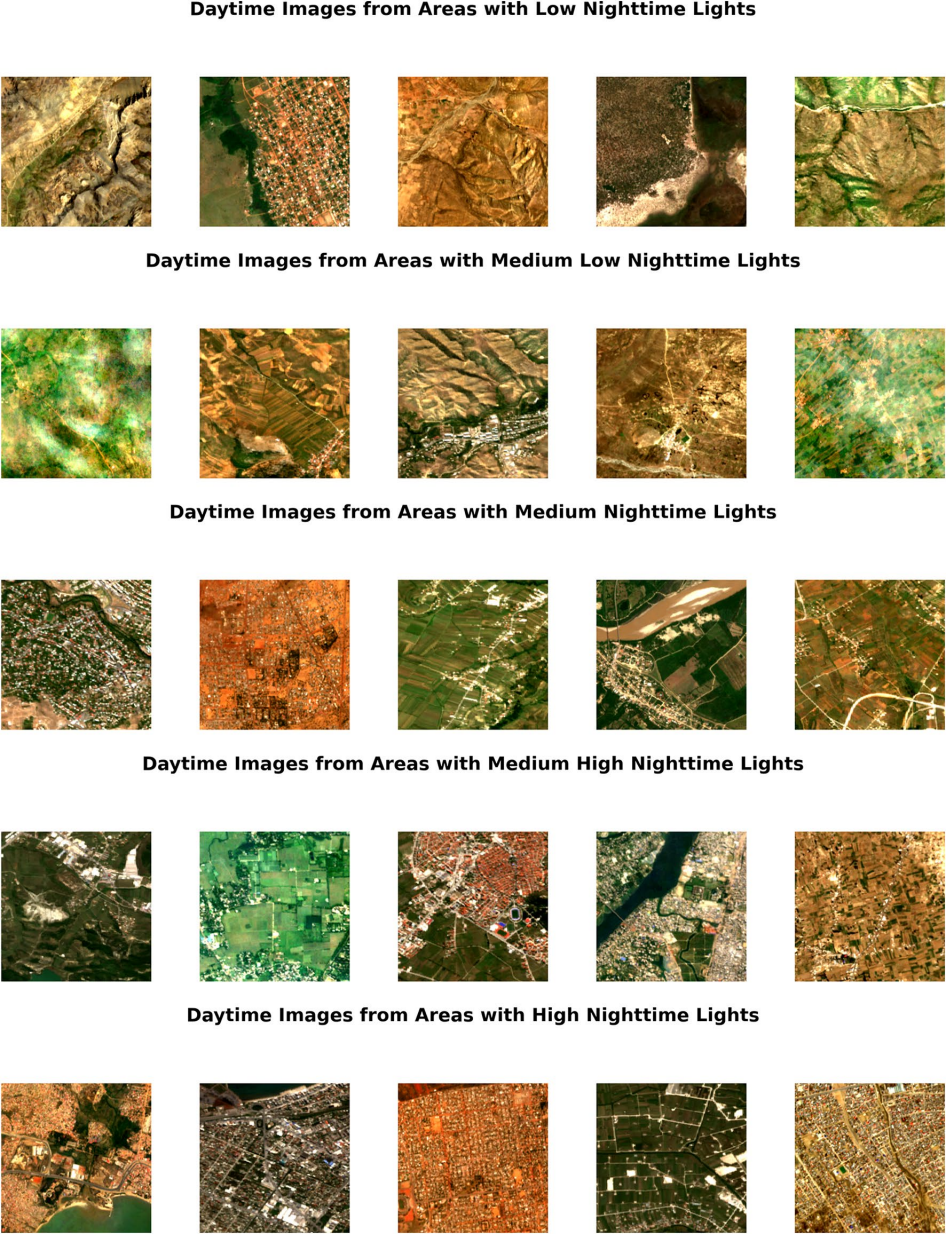
Example daytime images from Sentinel-2 for different nighttime lights groups. The figure was produced using Python, version 3.9.13 (https://www.python.org/). Sentinel-2 data was queried using Google Earth Engine.

We use daytime imagery to predict nighttime lights. When estimating levels of wealth, we rely on VIIRS for nighttime lights data; when estimating changes, we rely on the DMSP-OLS harmonized dataset from^39^. We take average nighttime lights over the same time period as daytime imagery is taken. We then group nighttime light radiance into five equally sized groups, representing low to high nighttime lights. Figure 9 shows example daytime images from Sentinel across these five groups. For the CNN, we use the pre-trained VGG16 model and further train the model to predict nighttime lights.

For both Sentinel and Landsat data, we use tiles of 224 $\times$ 224 pixels, which is the tile size used to train the VGG16 model. Consequently, for estimating levels of nighttime lights, we use a daytime grid that is 2.4 $\times$ 2.4 km (224 * 10-m resolution), and we use average nighttime lights within 2.4 km of the survey cluster. For estimating changes in nighttime lights, we use a daytime grid that is 6.72 by 6.72 km (224 * 30-m resolution), and we use average nighttime lights within 6.72 km of the survey cluster.

We train three separate CNN models using different sets of daytime imagery. First, following typical approaches, we train a model using the visible red, green, and blue bands. For the second and third models, we take advantage of Landsat and Sentinel capturing multiple spectral bands beyond those visible to the human eye. We compute two indices using multiple bands: the normalized difference vegetation index (NDVI), a common metric for capturing biomass or vegetation, and the built-up index (BU), a metric to capture human settlement or built-up areas^43^. NDVI and BU are computed as:

1 $$\begin{aligned} NDVI & = \frac{NIR - Red}{NIR + Red} \end{aligned}$$

2 $$\begin{aligned} BU & = \frac{SWIR - NIR}{SWIR + NIR} - NDVI \end{aligned}$$

where NIR is the near-infrared band and SWIR is the shortwave infrared band. Our second CNN model uses NDVI as an input and our third model uses BU as an input. The pre-trained VGG16 model requires three layers of inputs; consequently, we repeat the NDVI and BU bands into three layers in order to still take advantage of the pre-trained model

#### MOSAIKS

In addition to implementing CNN models to extract features from satellite imagery, we also leverage the MOSAIKS platform; MOSAIKS was created to simplify the process of generating informative features from high-resolution satellite imagery^19^. MOSAIKS generates random convolutional features using satellite imagery from Planet. The MOSAIKS platform allows for extracting features for any location, allowing users to then train machine learning models using these features. The MOSAIKS API outputs 4000 features for each location, where each location represents about a 1 km grid. We extract these features for all DHS survey locations. To reduce dimensionality of the data, we take the principal component of MOSAIKS features and keep components that explain 99.9% of the variance.

#### Daytime imagery

In addition to relying on CNN models to extract features from daytime imagery, we also test a more simple use of daytime imagery: extracting the average and the standard deviation of values from imagery. We take values of spectral bands within 2.5 km buffers. We rely on Landsat 7 when estimating levels and changes. Landsat 7 captures daytime imagery across six different spectral bands (e.g., red, green, blue, and near-infrared imagery), where data from most spectral bands are captured at a 30 m resolution. We take the average and standard deviation of values from each spectral band near each survey location; as with nighttime lights, we take the average using imagery from the years before, during, and after the survey was implemented. In addition to using the raw values from the spectral bands, we also use average values of NDVI and BU. NDVI and BU are more interpretable metrics than the individual bands and have a more expected relation with wealth. For example, more built-up (urban) areas may see greater wealth than rural areas. However, while the individual bands do not provide as interpretable metrics, the bands and combinations can capture factors related to wealth.

#### Facebook advertising data

Fatehkia et al.^44^ demonstrate the use of Facebook advertising data for wealth estimation using data from the Philippines and India. They use the Facebook Marketing API to query the number of Facebook monthly active users that match certain criteria, such as the number using certain devices (iOS vs. Android) and the use of high-end devices (latest iPhone or Samsung Galaxy Phones).

Following^44^, we query the number of monthly active Facebook users (MAUs) across a variety of attributes around each survey location. We query 34 different attributes, from categories including reported interests (e.g., interest in online shopping), behaviors (e.g., frequent travelers), and how one accesses Facebook (e.g., through an expensive phone like a new iPhone). We compute the proportion of Facebook users across all attributes. The Facebook Marketing API allows for querying areas with a minimum 1 km radius, and to protect privacy does not provide MAU values less than 1000; consequently, areas with low Facebook usage are reported to have an MAU of 1000. In addition to querying attributes, we also estimate the proportion of the population near the survey cluster on Facebook; to estimate this, we divide the MAU by population determined from WorldPop—which provides gridded population estimates^45^.

Choosing the radius to query Facebook data requires balancing the radius being large enough to have sufficient Facebook users (i.e., over 1000) but not extending too far beyond the survey cluster location. Masoomali et al.^44^ use a 2 km radius for urban areas and a 5 km radius for rural survey locations in the Philippines and—to account for lower Facebook penetration in India—use larger radii of 5 km and 10 km for urban and rural locations in India. We use an alternative, data-driven approach to define the radius for querying data both because (1) a goal of poverty estimation is to extrapolate to areas beyond survey locations, and we may not necessarily know whether a location should be classified as urban or rural; and (2) we query data from a larger set of countries which would make defining custom radii for each country cumbersome.

To choose the radius to query Facebook data, for each survey location, we check MAU of all Facebook users using a radius of 2 km, 5 km, and 10 km. We use the smallest radius that has at least 2000 MAUs (if none have 2000 MAUs, we use the 10 km radius). We use a threshold of 2000 MAUs as we are interested in the proportion of Facebook users across characteristics; using a threshold above 1000 for all Facebook users helps to ensure that MAUs of Facebook users across select characteristics also have a value above 1000. 2 km, 5 km and 10 km radii follow from the random displacement of DHS location displacement values; urban clusters are displaced up to 2 km, and most rural clusters are displaced up to 5 km—with 1% displaced up to 10 km. Our strategy for choosing radii to query Facebook for each cluster allows urban clusters to use a 5 km or 10 km if there is low Facebook penetration. In addition, our strategy also allows a rural cluster to use a 2 km radius; here, there is a chance that the radius chosen for Facebook does not cover the true DHS cluster location. However, given that there is likely some degree of positive spatial autocorrelation of poverty, if the queried location does not capture the true cluster location, the queried values likely still provide values that would be roughly similar to those at the true cluster location.

#### OpenStreetMap

OpenStreetMap (OSM) is a crowdsourced initiative to create a global geographical database^46^. OSM contains data on roads, buildings, points of interest (e.g., schools and heath facilities) and other features such as bodies of water. Existing research has incorporated measures from OSM into poverty estimation models as features such as accessibility to different road types and points of interest can be associated with socioeconomic levels^7,12,47,48^. For example, more wealthy areas may be both situated closer to services such as health clinics and schools and have better access to services through a large road network. In addition, the number of points of interest and the size of the road network near survey locations can also help indicate the sparsity or density of the location.

We adapt strategies for extracting features from OSM data from existing work, particularly^7^. We first extract the number of each type of point of interest near each survey location (within 2.5 km) and the minimum distance to each point of interest. OSM contains hundreds of types of points of interest; we use the 50 most common points of interest, determined by summing the number of points of interest near each survey location across all survey locations. Second, we extract the length of each road type near each survey location and compute the minimum distance to each road type. Road types include motorways, trunk, primary, secondary, tertiary, service, and pedestrian.

We only use OSM data to measure levels of wealth. Historic OSM data can be downloaded, so OSM could potentially also be used to explain changes in wealth. However, changes in OSM data could be due to both (1) new roads or points of interest or (2) OSM data being updated to better reflected currently existing roads or points of interest. More recent changes in OSM may more reflect new roads or points of interest. However, as OSM was founded in 2006 and many of the first round DHS surveys in our dataset come from the early 2000s, changes from the early 2000s to near the present may more reflect new data simply being entered into OSM. Consequently, we only use OSM data when estimating levels of wealth.

#### Land cover and characteristics

Land cover characteristics provide a useful context for understanding socioeconomic characteristics. For example, land cover can indicate the extent of urban and cropland areas. We use the European Space Agency Climate Change Initiative Land Cover dataset, which is available annually from 1992 to 2018 at 300 m resolution^49,50^. It classifies each pixel into one of the 36 land cover classes defined by the United Nations Land Cover Classification System (e.g., build-up/urban, cropland, etc). We extract the proportion of land classified according to each class near each survey cluster. In addition, following^7^, we include physical land characteristics including elevation and slope. We use slope and elevation data from the NASA Shuttle Radar Topography Mission (SRTM), which provides imagery at a 30 m resolution.

#### Climate and weather

Climate and weather contribute to food scarcity and agricultural production, particularly in rural areas. Following from^7^, we include long-term climate features from the WorldClim version 2 dataset, which captures annual trends, seasonality, and extreme values of temperature and precipitation at a roughly 1 km resolution; values are averaged from data from 1970 to 2000^51^. In addition to long-term climate trends, we include temperature and precipitation features captured the year the survey was implemented from the Copernicus Climate Change Service^52^.

#### Pollution

Previous research has shown that in many geographic regions, poorer areas tend to be associated with higher levels of air pollutants^26^. Following this literature, we use measures of pollution around survey locations from two data sources. First, we use pollution measures from Sentinel-5 Precursor (Sentinel-5P), a satellite designed specifically to monitor the Earth’s atmosphere launched in 2017^53^. From Sentinel 5-P, we use pollution metrics including Nitrogen Dioxide, a UV aerosol index, Carbon Monoxide, Formaldehyde, Ozone, and Sulfur Dioxide. Together, these sources capture pollution-generating activities such as fossil fuel and biomass burning, and traffic and industrial activity. We use average pollution measures captured from 2018 to 2020. As a second source of pollution data, we use a measure of aerosol optical depth (AOD) from the Moderate Resolution Imaging Spectroradiometer (MODIS), which provides imagery since 2000^54^. AOD is a measure of the amount of aerosols in the atmosphere, and has been shown to correlate strongly with measures of particulate matter^55^.

#### Synthetic aperture radar data

We use synthetic aperture radar (SAR) data from Sentinel-1. SAR data is based on transmitting waves and measuring the strength and orientation of waves reflected back to the satellite sensor^21^. As SAR sensors both transmit and receive signals, SAR data is unaffected by cloud cover—unlike daytime and nighttime satellite imagery which receives optical data reflected by the earth’s surface. Different objects scatter waves differently. For example, buildings and metallic objects produce a strong signal back to the sensor, and flat surfaces produce a weak signal^22^. Research has used SAR data for uses such as vehicle detection, crop classification, and urban change monitoring^22–24,56,57^.

We use the average and standard deviation of VV and VH bands, and compute the average and standard deviation of VV/VH. VV indicates waves that are transmitted vertically and received vertically, and VH indicates waves that are transmitted vertically and received horizontally. We use a 2.5 km buffer to compute values. Using all three metrics—VV, VH, and the ratio of the two—helps to further distinguish objects or land types. For example, vertical objects near smooth surfaces will give a strong VV signal but a weak VH signal^58^.

## Machine learning models

We train four different machine learning algorithms that are well suited to handle high-dimensional data: the Extreme Gradient Boosting (XGBoost) algorithm, regularized linear regression (lasso, ridge, and elastic net), and support vector machines^28–30^. We test four approaches for defining train and test sets:

- *Within country estimation* We train models only using data within each country. We divide the country into five folds, training the model on four folds and predicting wealth on the remaining fold. Folds are created by randomly assigning each second administrative division to one of the five folds; the survey cluster is assigned the fold by using the administrative division it falls in. We use the Database of Global Administrative Areas (GADM) for the boundaries of administrative divisions. In cases where the second administrative level is not available, we rely on the first administrative unit; this occurred in four cases (Armenia, Comoros, Lesotho, and Moldova). In one case (Comoros) we use three folds as there were only three administrative divisions. We create folds using administrative divisions to mitigate against nearby survey clusters being both in the train and test sets.
- *Within continent estimation* For each country, we train a model using all other countries in the same continent to predict wealth in the country. Given that some continents only have a few countries (Europe has two countries with data available from DHS), we use the following continent groupings: (1) Americas (eight countries); (2) Euroasia (two countries in Europe and thirteen countries in Asia); and (3) Africa (36 countries).
- *Other continents estimation* We train a model using each pair of continents and predict wealth in the other continent.
- *Global estimation* For each country, we train a model on all other countries to predict wealth in the country.

The approaches allow testing the balance between using data in a similar context to extending training data to using more data but in less similar contexts. At one extreme, we rely only on data within each country to train a model for wealth estimation; this approach uses the least amount of data, but helps ensure the training set comes from a similar context as the test set (while still ensuring that training and test sets do not spatially overlap). At the other extreme is leveraging data on all countries to predict wealth in a single country; while this approach takes advantage of using more data, it may suffer from introducing training data that may be less helpful for predicting wealth in a specific context (e.g., training with data on Armenia may not be helpful for predicting wealth in Lesotho). Training with data from other countries within the same continent provides a middle-ground approach. Training with data from countries in different continents directly tests whether training with data on less similar countries can still provide useful wealth estimates.

Separately testing these four approaches and selecting the approach that works best could lead to overfitting. Overfitting could result as a test set is held out separately for each approach. Consequently, throughout the paper, we do not analyze results based on the best performing approach—or the best performing approach for each country. We include analysis that compares results across the four approaches; for the remaining analysis, we use the global estimation approach as our default model—which ensures that the train and test sets are geographically dissimilar and leverages the most amount of training data.

Each algorithm has hyperparameters that can be tuned to improve model performance. To tune hyperparameters, we use a nested cross-validation approach^59^. After dividing data into test and train sets, we further divide each training set into five folds. Within the training set, we train the model using each set of hyperparameters on four folds and test model performance on the fifth fold; this process is repeated across all folds. We use the hyperparameters that produced the highest average model performance across folds to train the model on the full training set. This process ensures that the test set is not used for hyperparameter tuning; a separate cross-validation approach is applied only within the training set to select the optimal hyperparameters. For the XGBoost algorithm, we tune on the number of decision trees trained (50 and 100), the maximum depth of the tree (2 and 6), and the proportion of the training data randomly sampled prior to growing trees (0.5 and 1); each combination of parameters is used. For regularized linear regression we tune the ElasticNet mixing parameter using 0 (ridge regression), 1 (lasso regression), and 0.5 (elastic net), and we tune the lambda penalty using 10 values from 0.000001 to 5 on an increasing log scale. For support vector machines, we tune the cost *C* parameter (1, 5, and 10).

Appendix S11 compares model types across algorithms. For levels, on average, the XGBoost algorithm performs best. For changes, all algorithms perform similarly; across all analyses, we rely on estimates from the XGBoost algorithm.

### Supplementary Information

Supplementary Information.

## Data Availability

Data to replicate all findings in the paper are available at https://github.com/dime-worldbank/big-data-poverty-estimation.

## Code availibility

Code to replicate all findings in the paper is available at https://github.com/dime-worldbank/big-data-poverty-estimation.
