# Supplementary material for: Global poverty estimation using private and public sector big data sources
Source: Sci Rep. 2024 Feb 7;14:3160. doi: 10.1038/s41598-023-49564-6 (PMC10850149; doi:10.1038/s41598-023-49564-6)
Supplement: Supplementary file 1 — Supplementary Information. [file 41598_2023_49564_MOESM1_ESM.pdf]

Appendices: Global poverty estimation using private and public sector big data sources

Contents

S1 DHS Summary Statistics S2

S2 Comparing DHS Wealth Index and Global Wealth Index S5

S3 Correlation of Facebook Features with Wealth Index for Each Country S6

S4 Within-Country Correlations of Top Features S7

S5 Scatterplots of True and Estimated Levels of Wealth for Each Country S8

S6 Estimating Levels of Wealth: Pooled Results by Continent S11

S7 Model Performance Estimating Levels of Wealth for Each Country and Feature Set S12

S8 Scatterplots of True and Estimated Levels Changes in Wealth for Each Country S14

S9 Explaining Error Variation S16

S10 Application: Estimating Wealth in Different Years using First Administrative Division S19

S11 Comparing Results Across Machine Learning Algorithms S21

S12 Variation in Wealth: Within and Across districts S22

S13 Comparison of Results to Other Papers S24

S14 Wealth asset index and consumption comparison S25

S15 Comparing DHS and Facebook education variables S28

S1 DHS Summary Statistics

Table S1 shows the 59 countries used for estimating levels of wealth and the survey year used, the number of survey clusters within each country, and the average and standard deviation of the global wealth index. Table S2 shows the same, but for the 33 countries used for estimating changes in wealth.

Table S1: DHS summary statistics of countries used for estimating levels of wealth

Country	DHS Year	N Clusters	Global Wealth Index Mean (sd)
Albania	2017	715	2.59 (0.68)
Angola	2015	625	-1.2 (1.4)
Armenia	2015	313	2.78 (0.54)
Bangladesh	2017	672	-0.34 (1.03)
Benin	2017	540	-1.01 (0.92)
Bolivia	2008	997	0.56 (1.41)
Burkina Faso	2017	224	-1.85 (0.88)
Burundi	2016	552	-2.14 (0.73)
Cambodia	2014	611	0.04 (1.17)
Cameroon	2018	430	-0.57 (1.25)
Chad	2014	624	-2.77 (0.75)
Colombia	2010	4868	1.88 (1.4)
Comoros	2012	242	-0.05 (0.99)
Congo - Kinshasa	2013	492	-2.81 (0.9)
Côte d'Ivoire	2011	341	-0.58 (1.14)
Dominican Republic	2013	524	1.52 (0.84)
Egypt	2014	1817	2.63 (0.62)
Eswatini	2006	270	-0.02 (1.46)
Ethiopia	2019	305	-1.95 (1.25)
Gabon	2012	332	-0.13 (1.33)
Gambia	2019	280	0.23 (1.19)
Ghana	2019	192	0.01 (0.78)
Guatemala	2014	853	0.99 (1.32)
Guinea	2018	401	-1.1 (1.37)
Guyana	2009	312	0.62 (1.14)
Haiti	2016	450	-1.39 (1.04)
Honduras	2011	1128	0.42 (1.39)
India	2015	28358	0.07 (1.25)
Indonesia	2002	1319	0.32 (1.28)
Jordan	2017	970	2.85 (0.72)
Kenya	2020	298	-0.86 (1.08)
Kyrgyzstan	2012	314	1.29 (0.7)
Lesotho	2014	399	-1.38 (1.09)
Liberia	2019	321	-1.84 (0.69)
Madagascar	2016	358	-2.25 (0.77)
Malawi	2017	148	-1.54 (1.1)
Mali	2018	328	-1.03 (1.16)
Moldova	2005	399	1.72 (1.12)
Morocco	2003	480	1.29 (1.76)
Mozambique	2018	221	-1.55 (1.37)
Myanmar (Burma)	2015	441	-0.62 (0.86)
Namibia	2013	550	0.15 (2.01)
Nepal	2016	383	-0.52 (1.01)
Niger	2012	476	-2.8 (1.12)
Nigeria	2018	1382	-0.5 (1.13)
Pakistan	2017	560	0.77 (1.35)
Peru	2009	1131	0.57 (1.57)
Philippines	2017	1213	0.61 (0.8)
Rwanda	2019	500	-1.62 (0.8)
Senegal	2019	214	-0.27 (1.23)
Sierra Leone	2019	557	-1.68 (0.98)
South Africa	2016	746	1.85 (1.45)
Tajikistan	2017	365	1.29 (0.93)
Tanzania	2017	436	-1.47 (0.99)
Timor-Leste	2016	455	-0.55 (1.17)
Togo	2017	171	-0.83 (0.88)
Uganda	2018	316	-1.9 (0.88)
Zambia	2018	535	-1.45 (1.44)
Zimbabwe	2015	400	-0.3 (1.79)

Table S2: DHS summary statistics of countries used for estimating changes in wealth

Country	1st Survey Closest to 2000			Latest Survey		
	Year	N Clusters	Global Wealth Index Mean (sd)	Year	N Clusters	Global Wealth Index Mean (sd)
Albania	2008	450	2.47 (0.75)	2017	715	2.67 (0.68)
Angola	2006	115	-0.86 (1.62)	2015	625	-0.81 (1.4)
Armenia	2010	308	2.57 (0.67)	2015	313	2.75 (0.54)
Bangladesh	1999	341	-1.35 (1.29)	2017	672	-0.55 (1.03)
Benin	2001	247	-0.94 (1.17)	2017	540	-0.73 (0.92)
Burkina Faso	1998	208	-1.41 (0.98)	2017	224	-1.05 (0.88)
Burundi	2010	376	-1.87 (0.92)	2016	552	-1.92 (0.73)
Cambodia	2000	470	-1.22 (1.01)	2014	611	0.16 (1.17)
Cameroon	2004	464	-0.81 (1.28)	2018	430	-0.32 (1.25)
Congo - Kinshasa	2007	293	-1.7 (1.06)	2013	492	-1.88 (0.9)
Côte d'Ivoire	1998	140	0.42 (1.4)	2011	341	-0.36 (1.14)
Dominican Republic	2007	1425	1.28 (1.04)	2013	524	1.69 (0.84)
Egypt	2000	998	1.67 (1.15)	2014	1817	2.6 (0.62)
Ethiopia	2000	535	-1.95 (0.86)	2019	305	-1.4 (1.25)
Ghana	1998	400	-0.72 (1.16)	2019	192	0.15 (0.78)
Guinea	1999	293	-1.31 (1.09)	2018	401	-0.7 (1.37)
Haiti	2000	316	-1.12 (1.22)	2016	450	-1.13 (1.04)
Jordan	2002	495	3.23 (0.43)	2017	970	2.86 (0.72)
Kenya	2003	399	-1.25 (1.32)	2020	298	-0.66 (1.08)
Lesotho	2004	381	-1.47 (0.74)	2014	399	-1.01 (1.09)
Liberia	2007	291	-1.81 (0.58)	2019	321	-1.59 (0.69)
Madagascar	2008	585	-1.35 (0.92)	2016	358	-1.43 (0.77)
Malawi	2000	560	-1.92 (0.79)	2017	148	-1.31 (1.1)
Mali	2001	399	-1.47 (1.12)	2018	328	-0.54 (1.16)
Mozambique	2009	270	-1.39 (1.18)	2018	221	-1 (1.37)
Namibia	2000	260	-0.14 (2.13)	2013	550	0.38 (2.01)
Nepal	2001	251	-1.41 (1.45)	2016	383	-0.51 (1.01)
Niger	1998	268	-1.72 (0.97)	2012	476	-1.5 (1.12)
Nigeria	2003	360	-0.39 (1.38)	2018	1382	-0.3 (1.13)
Pakistan	2006	957	0.04 (0.2)	2017	560	0.95 (1.35)
Peru	2000	1408	0.56 (1.82)	2009	1131	0.94 (1.57)
Philippines	2003	816	1.02 (1.35)	2017	1213	0.63 (0.8)
Rwanda	2005	456	-2.01 (0.62)	2019	500	-1.45 (0.8)
Senegal	2005	366	-0.57 (1.34)	2019	214	-0.04 (1.23)
Sierra Leone	2008	350	-1.59 (0.86)	2019	557	-1.38 (0.98)
Tajikistan	2012	343	1.24 (1.06)	2017	365	1.52 (0.93)
Tanzania	1999	173	-1.72 (0.93)	2017	436	-1.32 (0.99)
Timor-Leste	2009	454	-1.24 (1.08)	2016	455	-0.36 (1.17)
Togo	1998	287	-1.13 (0.82)	2017	171	-0.63 (0.88)
Uganda	2000	267	-1.61 (1.03)	2018	316	-1.36 (0.88)
Zambia	2007	319	-1.31 (1.36)	2018	535	-1.02 (1.44)
Zimbabwe	1999	221	-0.47 (1.78)	2015	400	-0.06 (1.79)

S2 Comparing DHS Wealth Index and Global Wealth Index

DHS provides an asset-based wealth index; however, the wealth index is not comparable across countries. We create a globally comparable wealth index by taking the first principle component of a set of asset variables across the entire dataset—pooled across countries and across time. Figure S1 shows the association between the original DHS wealth index and the global wealth index we create for each country. The indices are strongly associated across countries. 54 of 59 (92%) countries have an r^2 over 0.9, where the minimum r^2 between the the two indices is 0.36 in Albania.

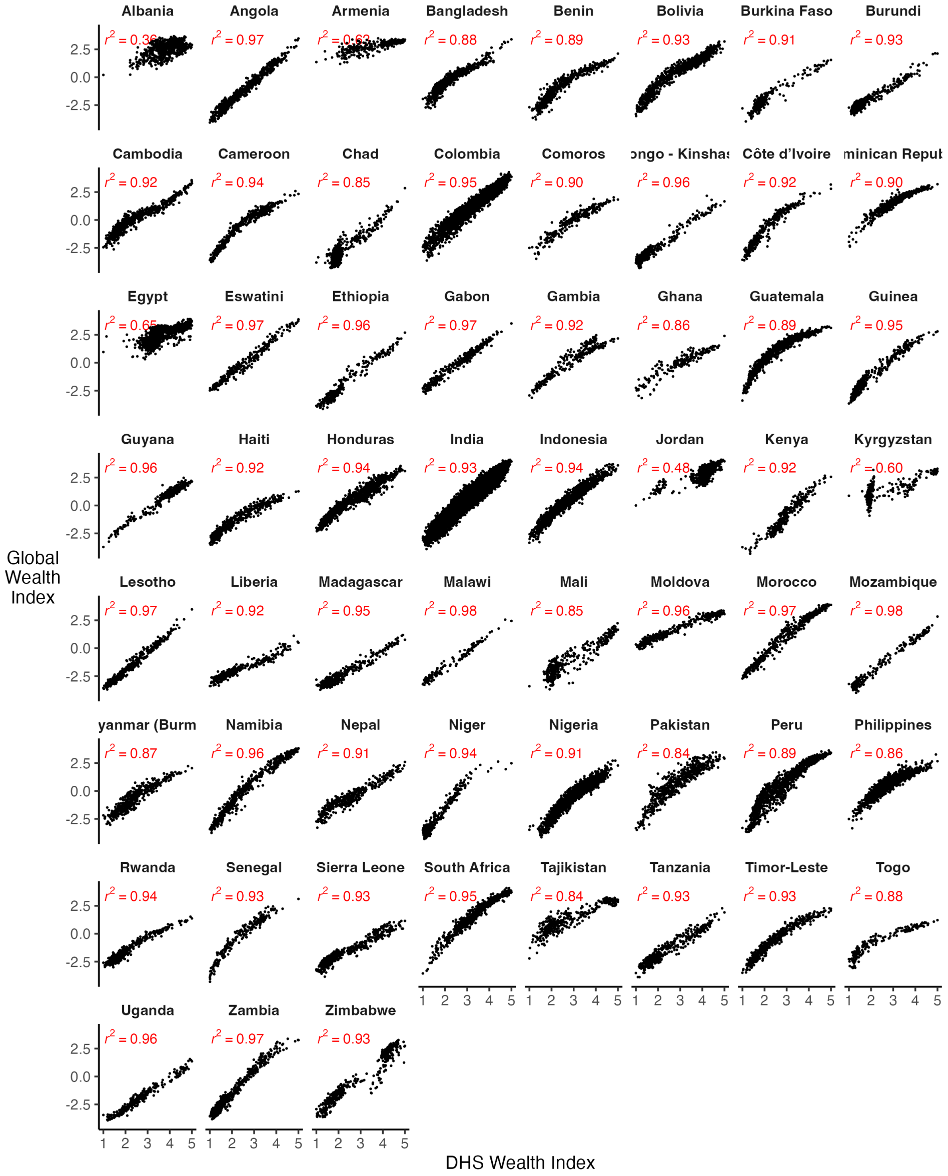

Figure S1: Association between DHS wealth index and global wealth index for each country

S3 Correlation of Facebook Features with Wealth Index for Each Country

Figure S2 shows the correlation coefficient for each feature from Facebook marketing data and the wealth index for each country. Many countries tend to see either low or high correlations across most variables. For example, most Facebook variables in Liberia have a correlation of 0.7 and above. Our data shows, though, that Liberia has low Facebook penetration: 14% of the population was active in the month where we queried data, compared to the median of 33% across the 59 countries. In a country with lower Facebook penetration, the variables may be highly correlated with wealth due to just indicating the presence of any active Facebook users in the location—where the presence of Facebook users may be indicative of higher wealth. Figure S2 also illustrates which Facebook variables tend to see higher correlations with wealth across countries. In particular, the “interest” variables tend to see higher correlations with wealth.

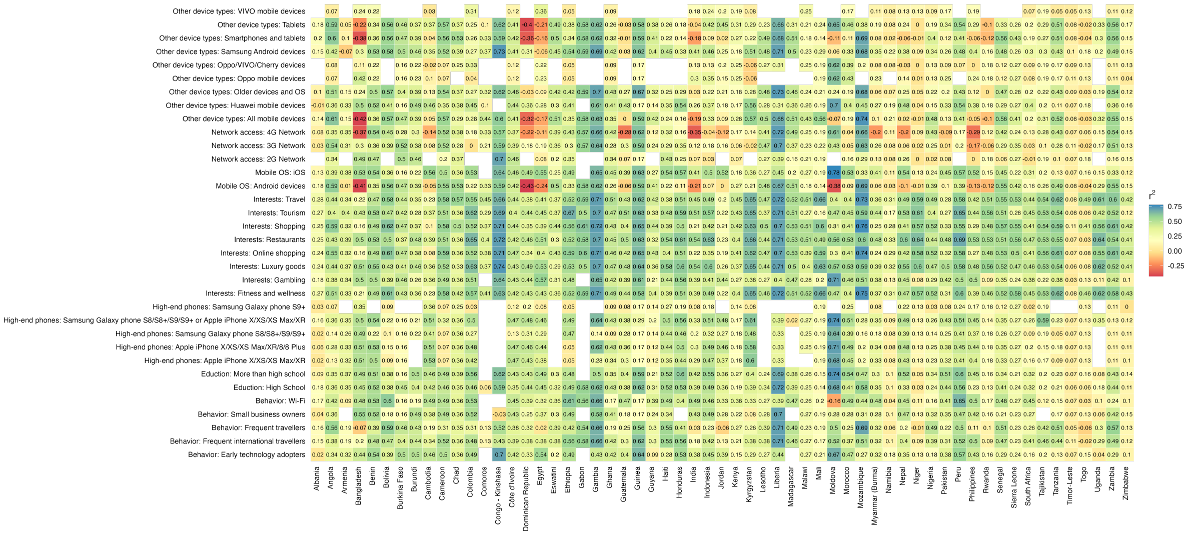

Figure S2: Correlation of Facebook features with wealth index

S4 Within-Country Correlations of Top Features

To understand the extent to which different variables across datasets capture the same dynamics, figure S3 shows the correlation between select features (panel A) and the standard deviation of correlations across countries (panel B). We use the feature with the highest correlation with the DHS wealth score for each dataset. Many of the features most correlated with wealth also see high correlations with each other; nighttime lights, length of residential roads, and urban land cover all have more than a 0.7 correlation with each other. These features also see low standard deviations in correlations across countries, indicating the correlation between these features is strong across countries.

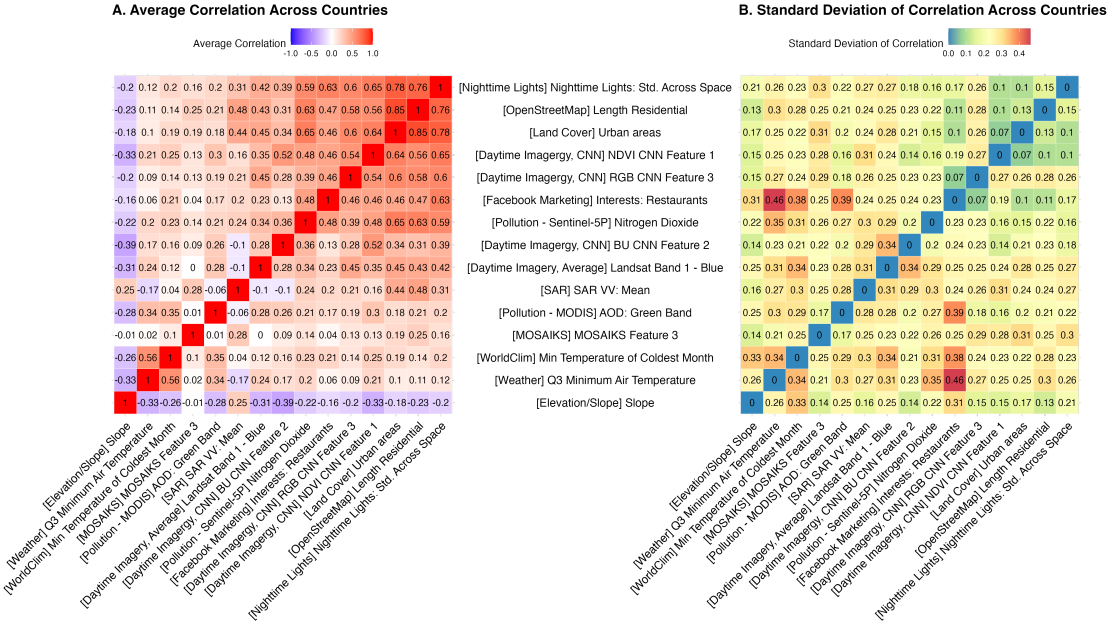

Figure S3: Correlation of variables between each other. We use the variable with the highest correlation to the wealth score in each dataset. **Panel A** shows the average correlation across countries and **panel B** shows the standard deviation in correlations across countries. The variables are ordered top to bottom and right to left according to their correlation with the wealth score.

S5 Scatterplots of True and Estimated Levels of Wealth for Each Country

Figure S4 shows the scatterplot of true and estimate levels of wealth for each country at the survey cluster level and figure S5 shows the same when aggregating data to the district level. The scatterplots illustrate the strong association between true and estimated wealth in many countries.

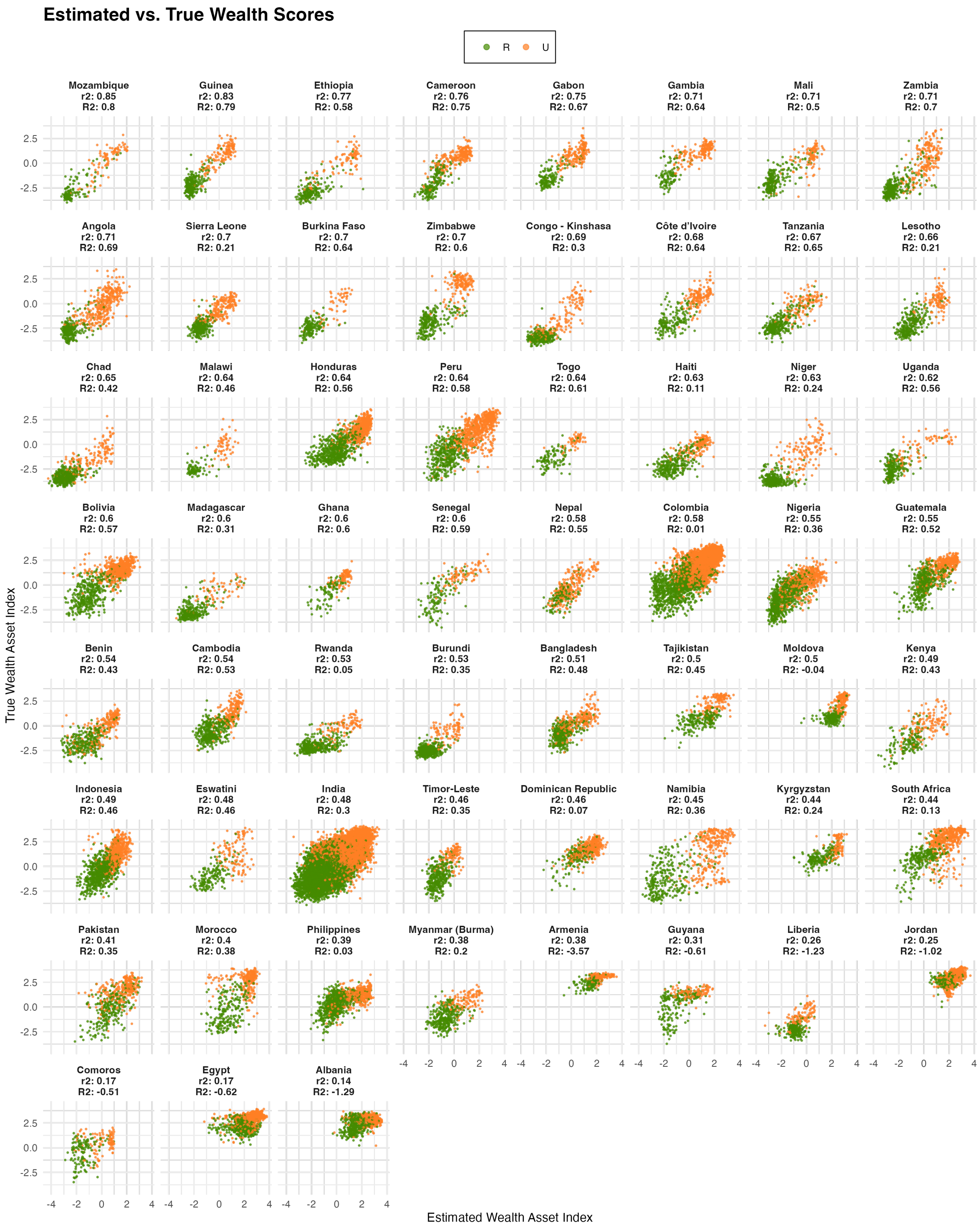

Figure S4: Scatterplot between true and estimated levels of wealth for each country using survey clusters as the unit analysis. r^2 is the squared Pearson correlation coefficient, and R^2 is the coefficient of determination.

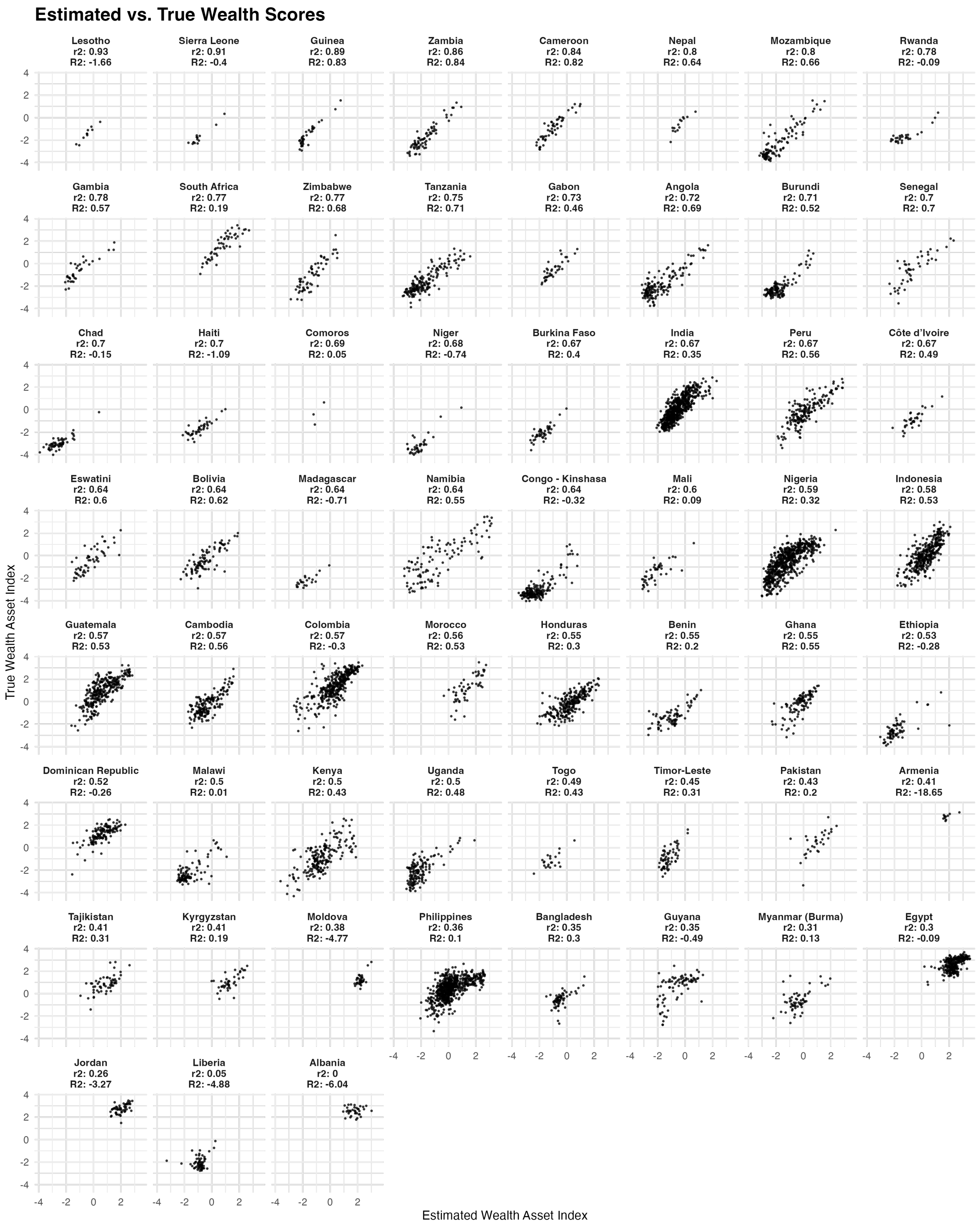

Figure S5: Scatterplot between true and estimated levels of wealth for each country when aggregating to districts. The r^2 is the squared Pearson correlation coefficient, and R^2 is the coefficient of determination.

S6 Estimating Levels of Wealth: Pooled Results by Continent

Figure S6 model performance when pooling results across countries, separated by continent. When pooling true and estimated wealth across countries, estimated wealth explains 75% of the variation in true wealth in Africa, 52% of the variation in the Americas, and 55% of the variation in Eurasia. Results at the district level are comparable.

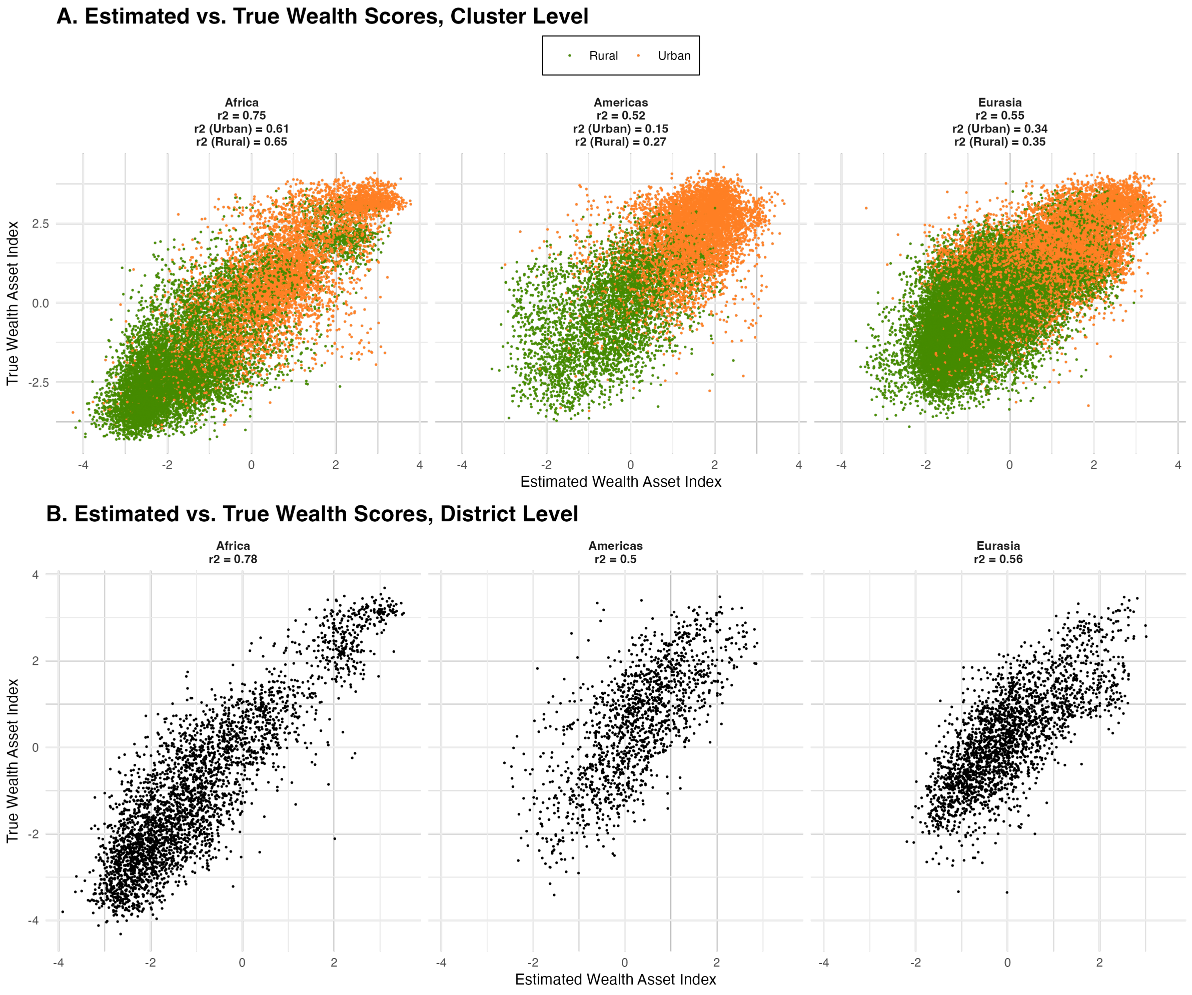

Figure S6: Scatterplot between true and estimated levels of wealth for each country when aggregating to districts. The r^2 is the squared Pearson correlation coefficient, and R^2 is the coefficient of determination.

S7 Model Performance Estimating Levels of Wealth for Each Country and Feature Set

Figure S7 shows model performance (r^2 between true and estimated wealth) for each country when training on each set of features. The figure illustrates variation across which feature sets work well across countries. Some countries are fairly consistent in most sets of features either working well or not working well. For example, no models trained on different sets of features work particularly well in Comoros, which indicates there may be something about the country or survey data that may result in the wealth estimation not working well.

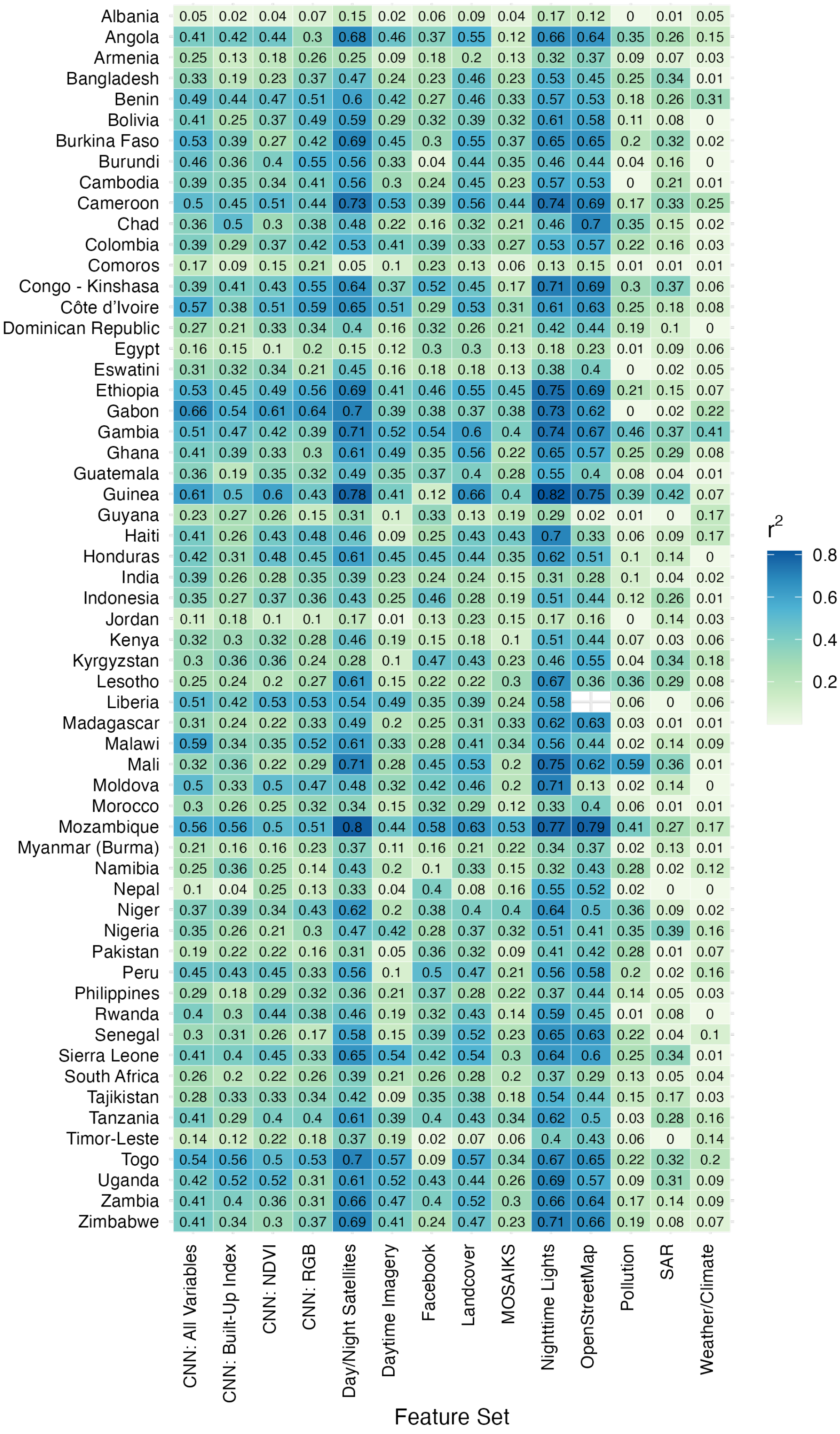

Figure S7: Model performance estimating levels of wealth for each country and feature set

S8 Scatterplots of True and Estimated Levels Changes in Wealth for Each Country

Figure S8 shows the scatterplot of true and estimated changes in wealth for each country at the survey cluster level and figure S9 shows the same when aggregating data to the district level.

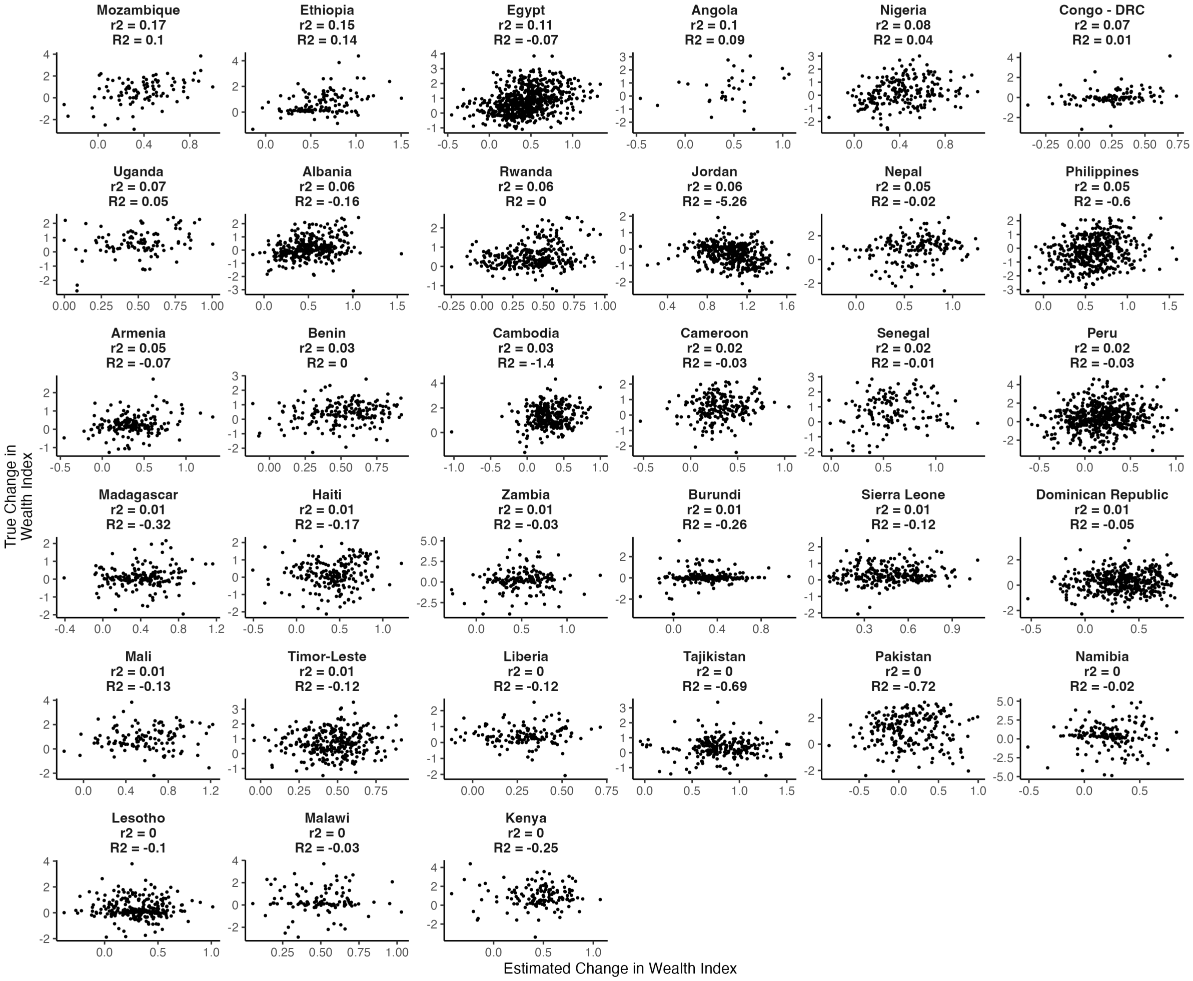

Figure S8: Scatterplots of changes in true and estimated changes in the wealth index at the cluster level. r^2 is the squared Pearson correlation coefficient, and R^2 is the coefficient of determination.

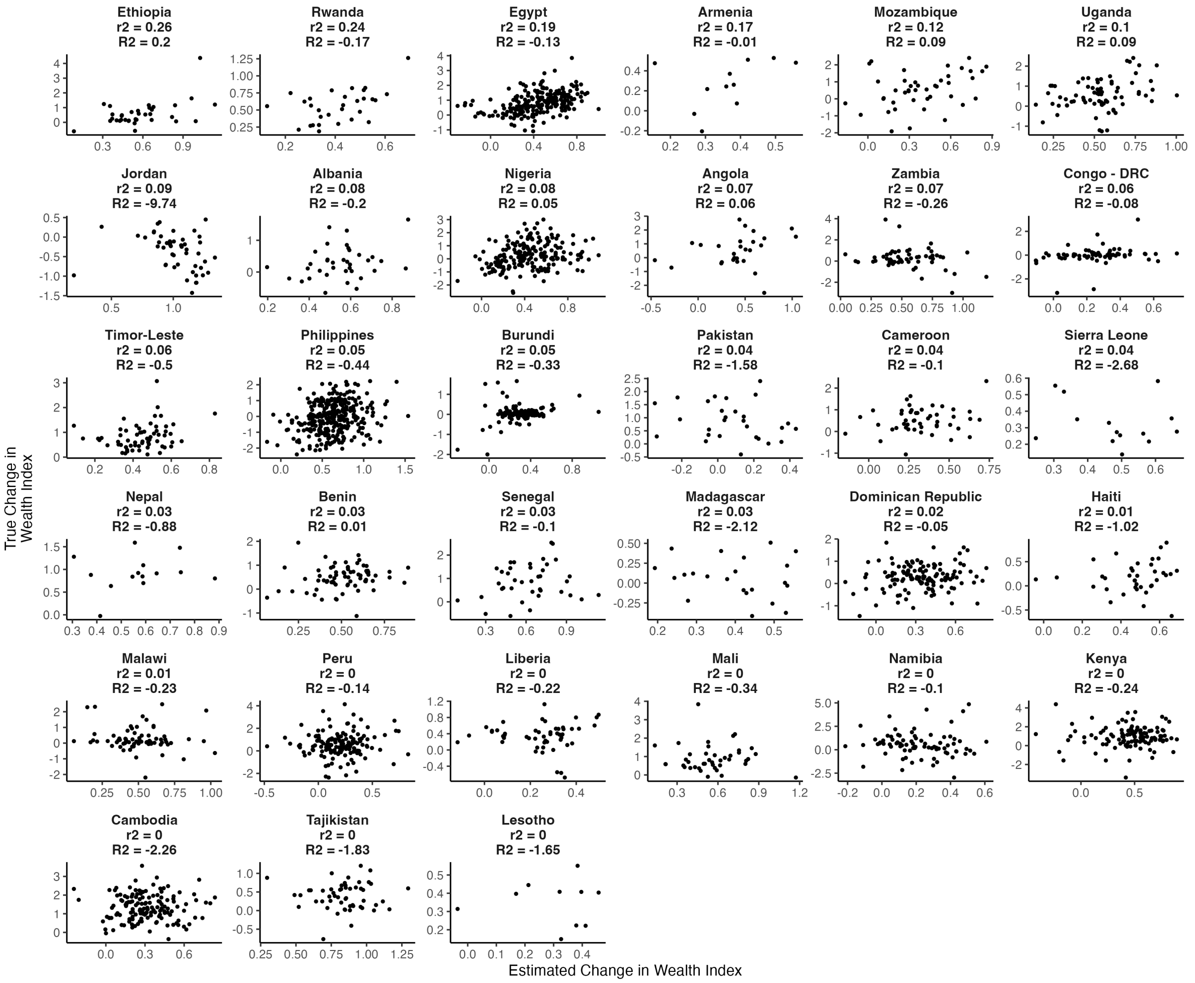

Figure S9: Scatterplots of changes in true and estimated changes in the wealth index at the district level. r^2 is the squared Pearson correlation coefficient, and R^2 is the coefficient of determination.

S9 Explaining Error Variation

For each DHS survey cluster, we compute the error between model prediction and true wealth. Here, we test whether the magnitude of this error (taking the absolute value of the difference between true and predicted wealth) is correlated with factors including nighttime lights, whether the cluster is classified as urban or rural, the geographic region, and country income level. We examine both errors for estimating levels of wealth and changes in wealth.

Tables S3 and S4 show results from regressing the error on the select factors. Figures S10 and S11 show results when examining each factor independently. For both levels and changes, error estimates tend to be slightly lower in rural locations, in Africa, and in low and lower middle-income countries compared to upper middle-income countries. For levels of wealth, when nighttime lights are low, there is a large variation in error; at the highest levels of nighttime lights, error estimates are low.

Table S3: Explaining error (absolute value of true minus predicted wealth) based on select factors

	Dependent variable:
	Absolute value of difference in true and estimated wealth
Nighttime lights	-0.126^{***}
	(0.003)
Urban	0.187^{***}
	(0.007)
Lower middle income	0.095^{***}
	(0.011)
Upper middle income	0.264^{***}
	(0.013)
Americas	0.062^{***}
	(0.011)
Eurasia	0.036^{***}
	(0.008)
Constant	0.785^{***}
	(0.009)
Observations	63,854
R^2	0.035
Adjusted R^2	0.035
Note:	*p<0.1; **p<0.05; ***p<0.01

Table S4: Explaining error (absolute value of true minus predicted wealth) based on select factors

	Dependent variable:
	Absolute value of difference in change in true and estimated wealth
Change in nighttime lights, absolute value	0.016***
	(0.001)
Urban (baseline)	0.169***
	(0.015)
Lower middle income	0.142***
	(0.021)
Upper middle income	0.158***
	(0.030)
Americas	0.094***
	(0.029)
Eurasia	0.191***
	(0.020)
Constant	0.409***
	(0.017)
Observations	7,714
R^2	0.094
Adjusted R^2	0.094
Note:	*p<0.1; **p<0.05; ***p<0.01

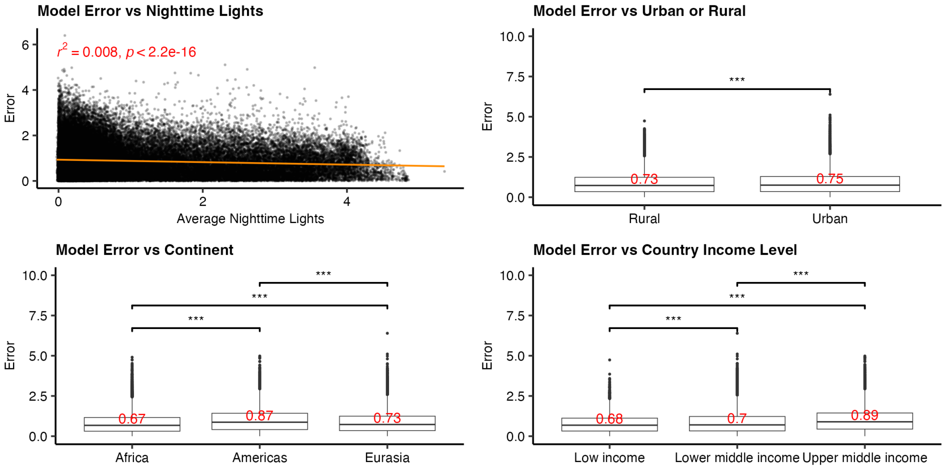

Figure S10: Explain error: levels. The boxplots include center line, median; box limits, upper and lower quartiles; whiskers, 1.5x interquartile range; points beyond whiskers, outliers. *p<0.1; **p<0.05; ***p<0.01

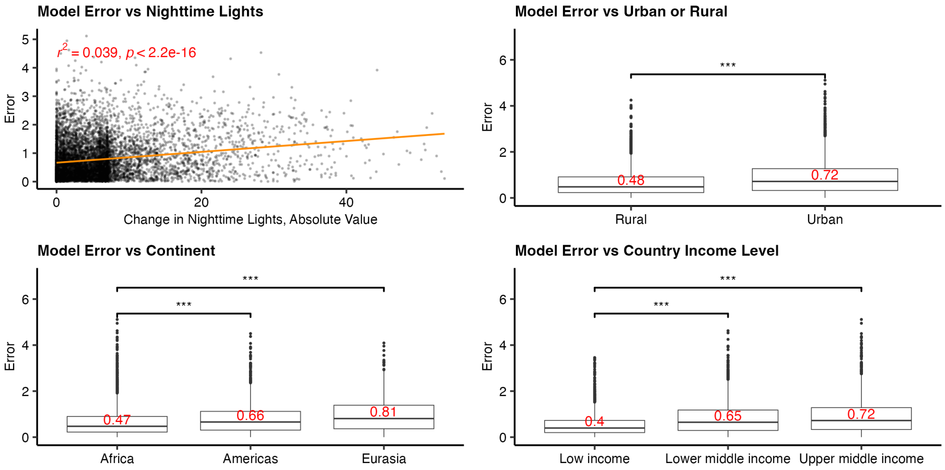

Figure S11: Explain error: changes. The boxplots include center line, median; box limits, upper and lower quartiles; whiskers, 1.5x interquartile range; points beyond whiskers, outliers. *p<0.1; **p<0.05; ***p<0.01

S10 Application: Estimating Wealth in Different Years using First Administrative Division

To illustrate the value added our the machine learning approach, we estimate wealth in Nigeria across different time periods—and compare estimates to interpolating or extrapolating wealth based on other DHS survey data. In the main text (see figure 9), we aggregate data to Nigeria's second administrative division. As a sensitivity analysis, figure S12 below shows results when aggregating data to Nigeria's first administrative division.

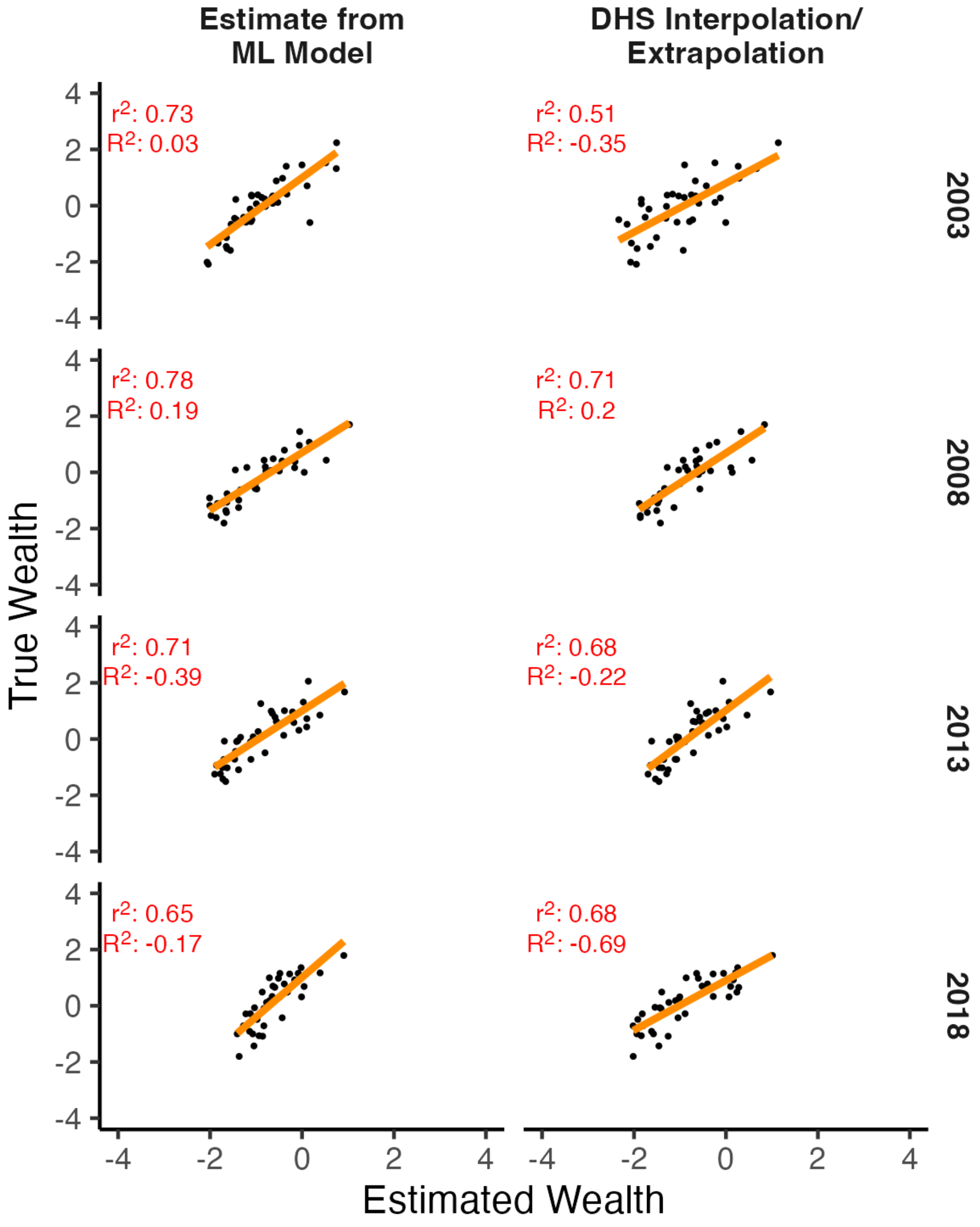

Figure S12: Comparison of true wealth estimates versus estimates from machine learning model and using DHS data to interpolate and extrapolate wealth estimates; data is extrapolated for 2003 and 2018 data, and interpolated for 2008 and 2013. For each year, the two closest DHS rounds are used to estimate wealth; for example, the data in 2003 and 2013 is used to interpolate wealth for 2008. Wealth estimates are at the first administrative division level.

S11 Comparing Results Across Machine Learning Algorithms

We test multiple machine learning algorithms: XGBoost, support vector machines (SVM), and regularized linear regression (lasso, ridge, and elastic net). Figure S13 shows the distribution of country-level results for explaining levels and changes of wealth, using models that are trained on all countries except the country of interest. For explaining both levels and changes in wealth, the median r^2 is highest using XGBoost.

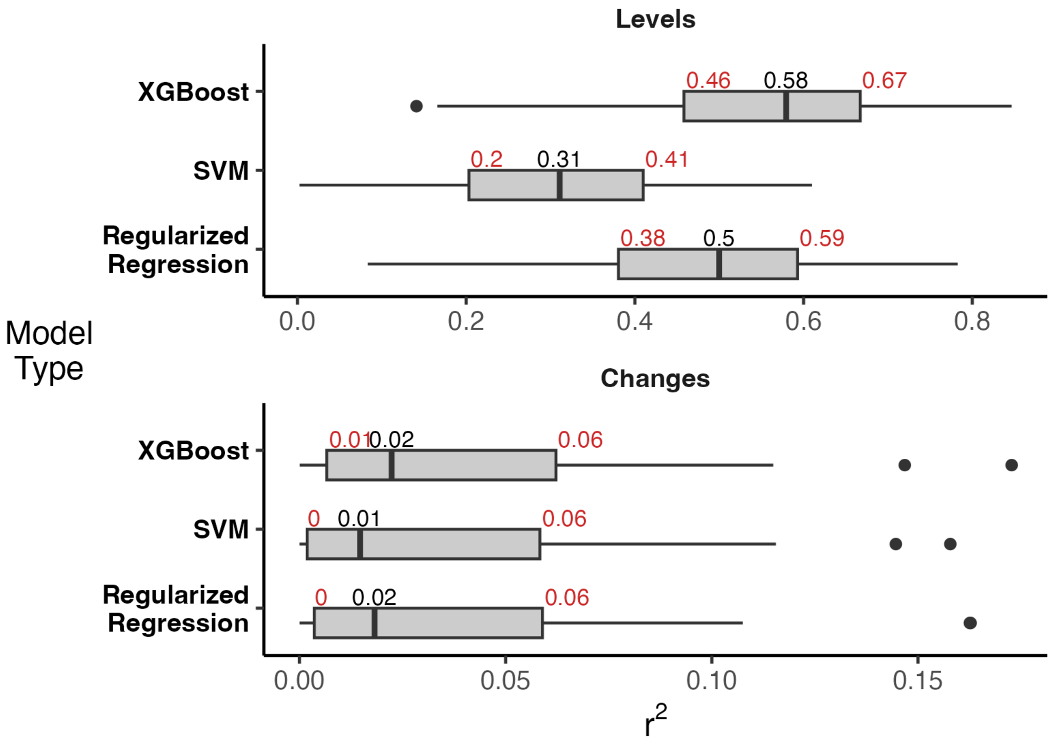

Figure S13: Comparison of model performance by machine learning algorithm. The boxplots include center line, median; box limits, upper and lower quartiles; whiskers, 1.5x interquartile range; points beyond whiskers, outliers.

S12 Variation in Wealth: Within and Across districts

Table S5 compares the standard deviation of wealth within districts and across districts for each country, using the wealth asset index. Out of the 59 countries, 35 (59%) have a larger standard deviation across districts compared to within districts.

Table S5: Comparing standard deviation in wealth within and across districts

Country	Std. Dev. Wealth Across Districts	Average Within District Wealth Std. Dev.
Albania	0.36	0.57
Angola	1.19	0.86
Armenia	0.22	0.42
Bangladesh	0.64	0.74
Benin	0.8	0.78
Bolivia	0.99	1.14
Burkina Faso	0.64	0.79
Burundi	0.79	0.47
Cambodia	0.93	0.62
Cameroon	1.05	1
Chad	0.55	0.65
Colombia	1.12	0.91
Comoros	0.99	0.98
Congo - Kinshasa	0.88	0.35
Côte d'Ivoire	0.73	1.31
Dominican Republic	0.57	0.64
Egypt	0.53	0.34
Eswatini	1.09	0.88
Ethiopia	0.92	0.79
Gabon	0.78	1.04
Gambia	1	0.75
Ghana	0.94	0.63
Guatemala	1.08	0.76
Guinea	0.97	0.97
Guyana	1.29	0.54
Haiti	0.62	0.84
Honduras	0.88	0.82
India	1.04	0.84
Indonesia	1.06	0.81
Jordan	0.41	0.36
Kenya	1	0.96
Kyrgyzstan	0.61	0.47
Lesotho	0.65	1.33
Liberia	0.56	0.47
Madagascar	0.49	1.05
Malawi	0.99	0.76
Mali	0.89	0.8
Moldova	0.44	0.71
Morocco	1.16	1.5
Mozambique	1.41	1.01
Myanmar (Burma)	0.83	0.84
Namibia	1.77	1.15
Nepal	0.7	0.98
Niger	0.88	0.82
Nigeria	1.16	0.71
Pakistan	0.9	1.19
Peru	1.16	1.21
Philippines	0.77	0.52
Rwanda	0.62	0.75
Senegal	1.23	1.13
Sierra Leone	0.75	0.8
South Africa	1.05	1.13
Tajikistan	0.7	0.61
Tanzania	1	0.72
Timor-Leste	0.87	0.91
Togo	0.66	0.82
Uganda	1	0.56
Zambia	1.13	1.23
Zimbabwe	1.29	1.24

S13 Comparison of Results to Other Papers

Our work builds off of many previous studies that estimate poverty or wealth. Table S6 compares results from our paper to three similar papers that also estimate wealth using DHS surveys [3, 1, 2].

Table S6: Comparison of results to other papers. Yeh et al. (2020) and Chi et al. (2022) use models trained on other countries to estimate wealth in each country of interest; consequently, when comparing our results to these papers, we show results from models using a similar approach—where models are trained on all other countries. For each country, Jean et al. (2016) trains models using only data for each country; consequently, when comparing our results to this paper, we show results from models using a similar approach—where models are trained on data within each country.

Unit	Level/Change	Aggregation	Location	Paper	Other Paper r^2	This Paper r^2
Village	Levels	Pooled	Africa	Yeh et al. (2020)	0.67	0.75
Village	Levels	Average across countries	Africa	Yeh et al. (2020)	0.7	0.60
Village	Levels	Pooled	Africa - Urban	Yeh et al. (2020)	0.4	0.61
Village	Levels	Pooled	Africa - Rural	Yeh et al. (2020)	0.32	0.65
District	Levels	Pooled	Africa	Yeh et al. (2020)	0.75	0.78
District	Levels	Average across countries	Africa	Yeh et al. (2020)	0.83	0.66
Village	Changes	Pooled	Africa	Yeh et al. (2020)	0.35	0.04
District	Changes	Pooled	Africa	Yeh et al. (2020)	0.43	0.05
Village	Levels	Average across countries	All DHS countries	Chi et al. (2022)	0.59	0.55
Village	Levels	Not applicable	Malawi	Jean et al. (2016)	0.55	0.48
Village	Levels	Not applicable	Nigeria	Jean et al. (2016)	0.68	0.70
Village	Levels	Not applicable	Rwanda	Jean et al. (2016)	0.75	0.58
Village	Levels	Not applicable	Tanzania	Jean et al. (2016)	0.57	0.69
Village	Levels	Not applicable	Uganda	Jean et al. (2016)	0.69	0.76

S14 Wealth asset index and consumption comparison

In this paper, we leverage an asset-based measure of wealth. An asset index approach is typically used when neither income nor expenditure data are available, as is the case with DHS data. To test the sensitivity of our results to using a measure of consumption, we leverage data for six countries using the sources of the World Bank poverty and inequality measures, the Living Standards Measurement Surveys (LSMS), available at http://pip.worldbank.org/home. LSMS data provide expenditures or consumption data used for the World Bank (monetary) poverty estimates and includes assets, which we used to construct an asset index using the same methods we use with the DHS data. Unlike DHS data, the surveys are not standardized, and LSMS does not publicly release GPS coordinates of survey clusters for all surveys.

For this analysis, we focus on sub-Saharan Africa where the method is more likely to be used, and for countries for which LSMS data is available around 2016–2019. We end up with the following sample of countries: Burkina Faso, Benin, Cote d'Ivoire, Ethiopia, Malawi, and Togo. Figure S14 shows the association between consumption and the wealth index for each country. The wealth index is positively and significantly associated with consumption and explains 40–66% of the variation in consumption depending on the country.

We retrain the machine learning model using data from LSMS, separately training the model to estimate the wealth index and consumption. We leverage the XGBoost algorithm to train models. For each country, we divide the country into five folds—where each fold is geographically separated—and use four folds to train a model to estimate asset wealth or consumption in the left-out fold.

Figure S15 shows that the model better estimates the asset-based wealth index compared to consumption in all countries. Despite differences in the wealth index and consumption, similar sets of features are most important in estimating both wealth indicators. Figure S16 shows the model performance when select sets of features are used to train the model. Models trained on nighttime lights, daytime and nighttime lights, and OpenStreetMaps perform best for both wealth indicators, while models trained on weather/climate features, SAR data, and Facebook features perform worse.

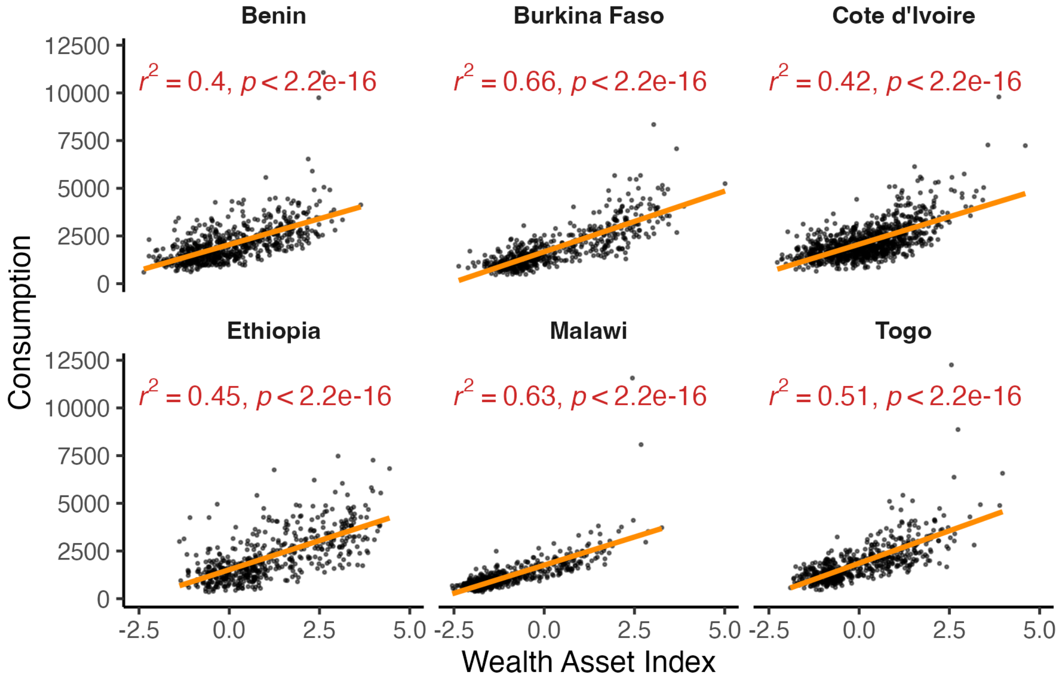

Figure S14: Comparison of wealth asset index and consumption

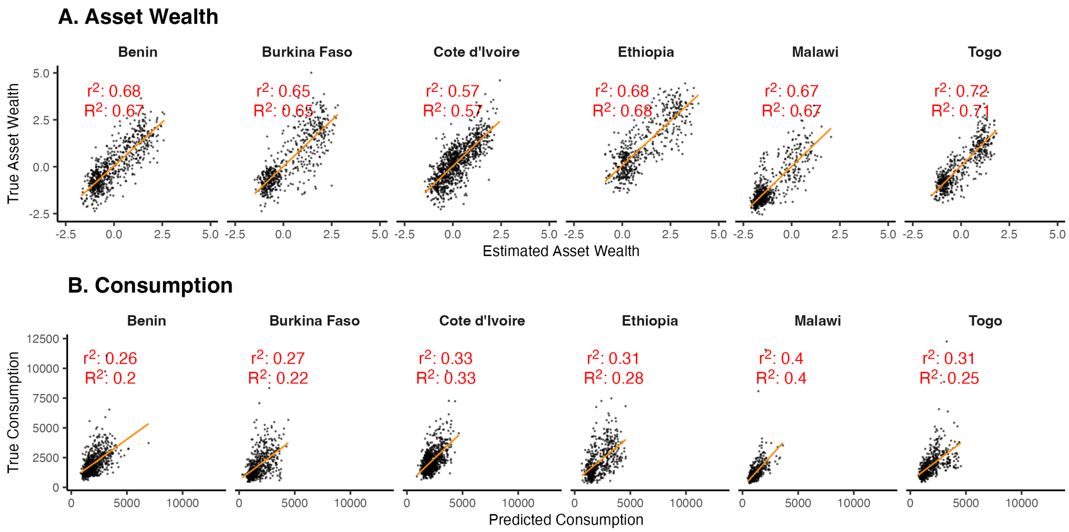

Figure S15: Comparison of model performance estimating wealth asset index and consumption

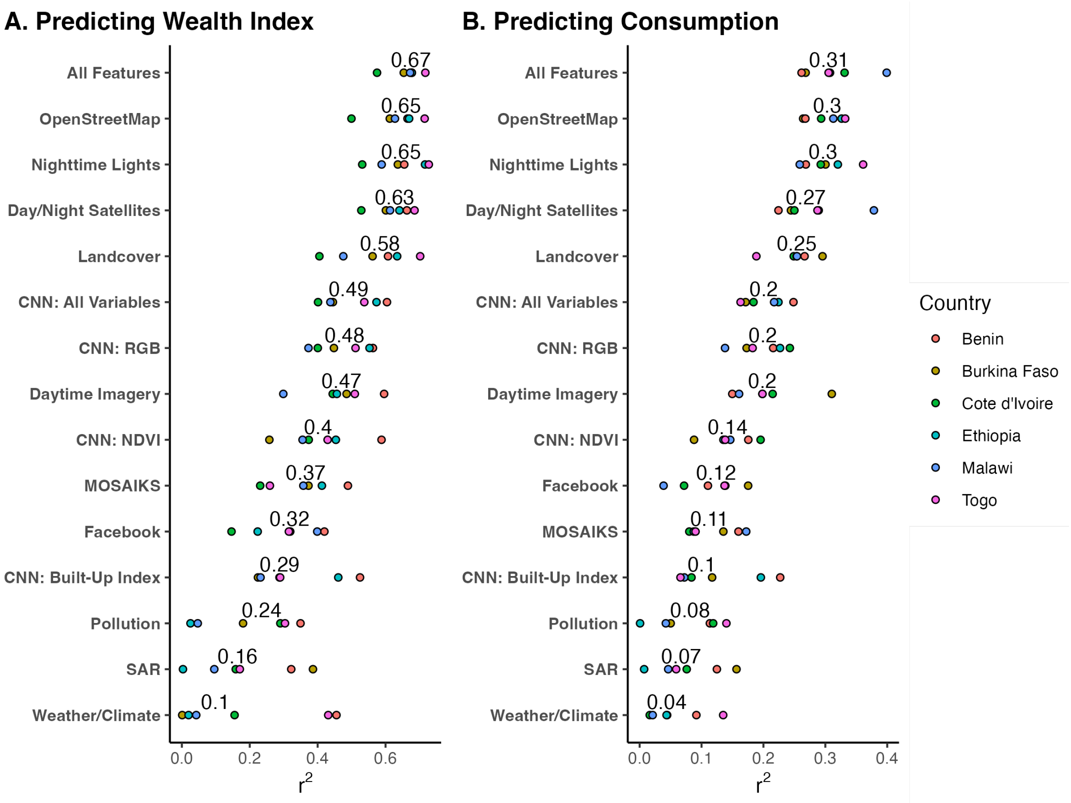

Figure S16: Comparison of model performance estimating wealth asset index and consumption by set of features used to train the model. The median r^2 is reported.

S15 Comparing DHS and Facebook education variables

The paper tests the ability of variables from Facebook Marketing data to estimate wealth. In this section, we test the ability of Facebook data to capture a similar variable from the Demographic and Health Surveys (DHS). We use a variable that is captured in both data sources: the proportion of those with higher than a high school education. From DHS, we use the proportion of household members in each cluster that has a higher than secondary education; from Facebook, we use the proportion of monthly active users that report having higher than a high school education. We estimate the correlation between the two variables both at the cluster and district levels, and restrict the analysis to countries with 30 or more districts.

Figure S17 shows the distribution of the within-country correlation at the cluster and district level. Correlation using both unit types has a large variation, with countries seeing both low and high correlations. However, the median correlation across countries at the cluster and district level is 0.41 and 0.53, respectively, showing that: (1) in most countries, above high school education captured by DHS and Facebook move roughly together; and (2) the correlation is larger at a higher aggregation. Figures S18 and S19 show scatterplots of the two variables across countries.

In figure S20, we attempt to explain the variation in correlation using (1) the number of units used to compute the correlation, (2) country population, and (3) the proportion of the population active on Facebook (relying on monthly active users for the month when the data from Facebook was queried). The figure shows no notable association between the within-country correlation and the country-level variables.

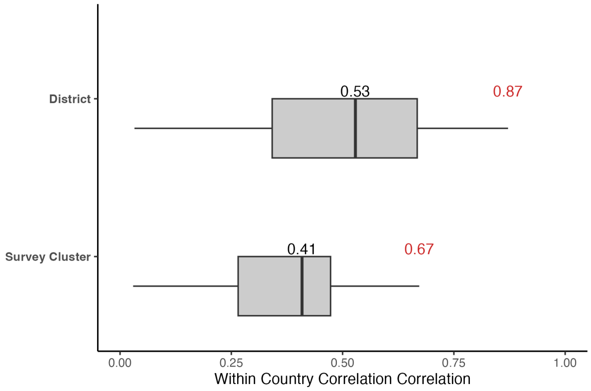

Figure S17: Distribution of within-country correlation between the proportion of the population with above high school education as measured by DHS and Facebook. The boxplots include center line, median; box limits, upper and lower quartiles; whiskers, 1.5x interquartile range; points beyond whiskers, outliers.

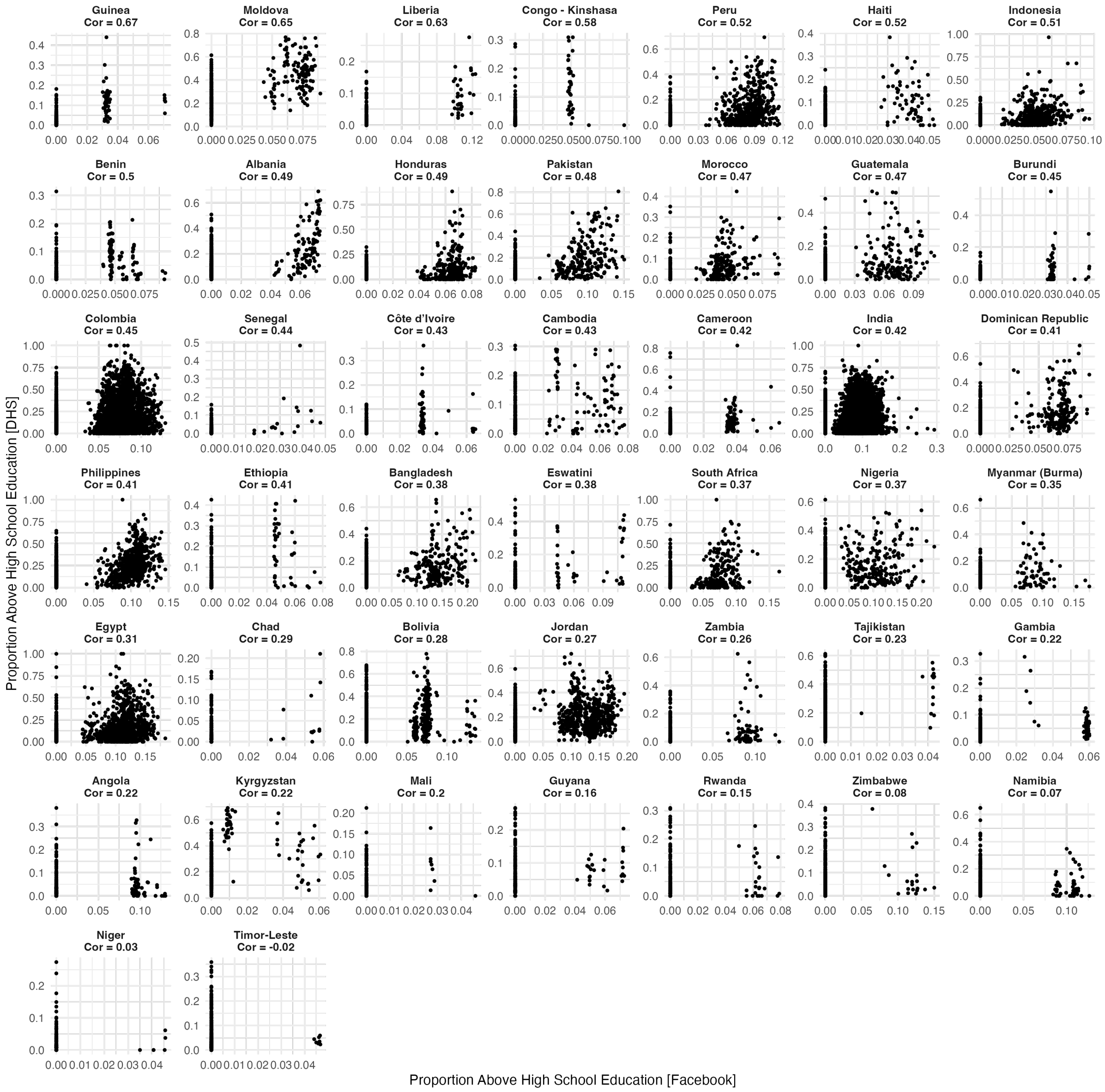

Figure S18: Cluster-level scatterplot between proportion with above high school education as measured by Facebook and DHS

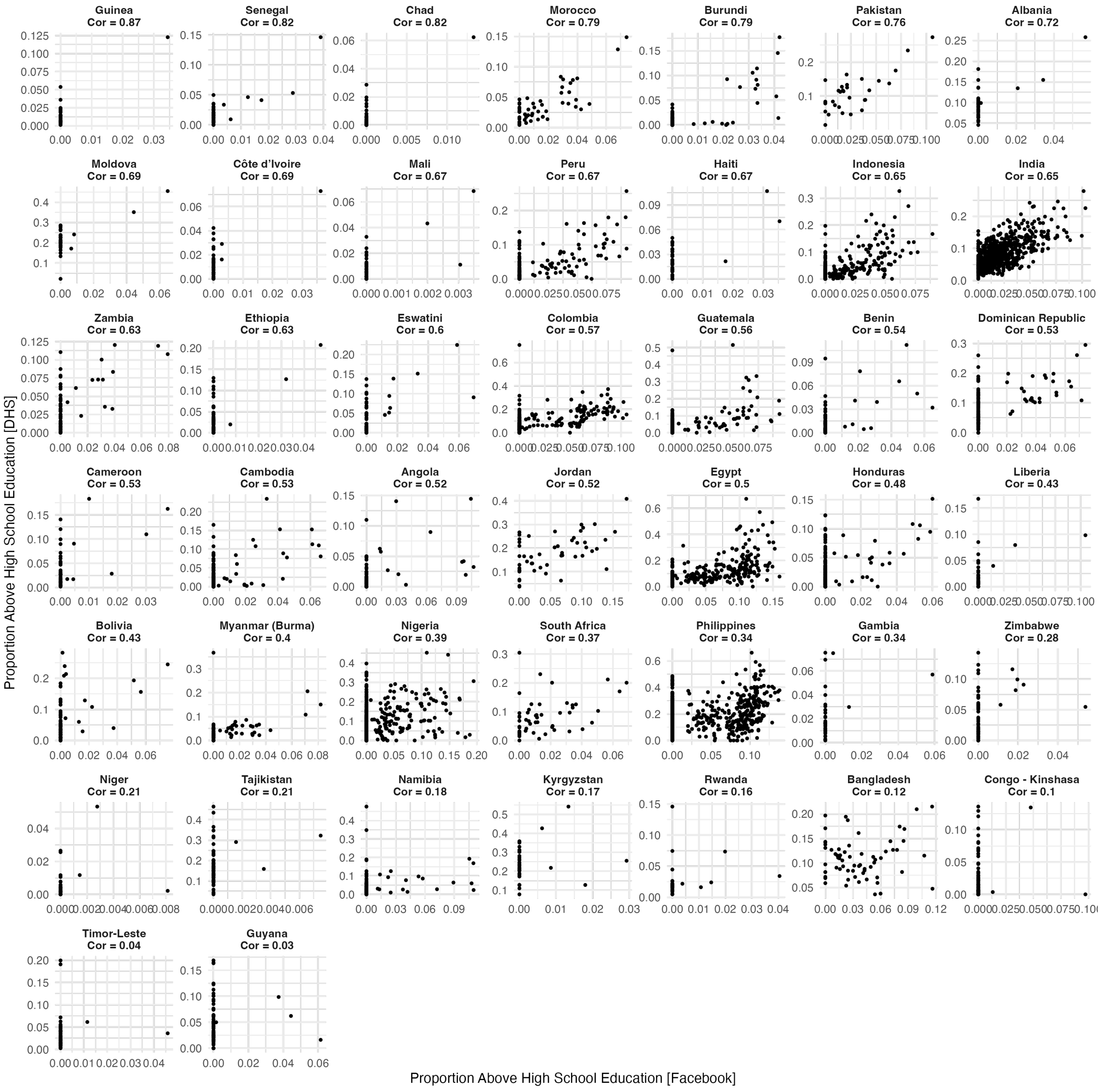

Figure S19: District-level scatterplot between proportion with above high school education as measured by Facebook and DHS

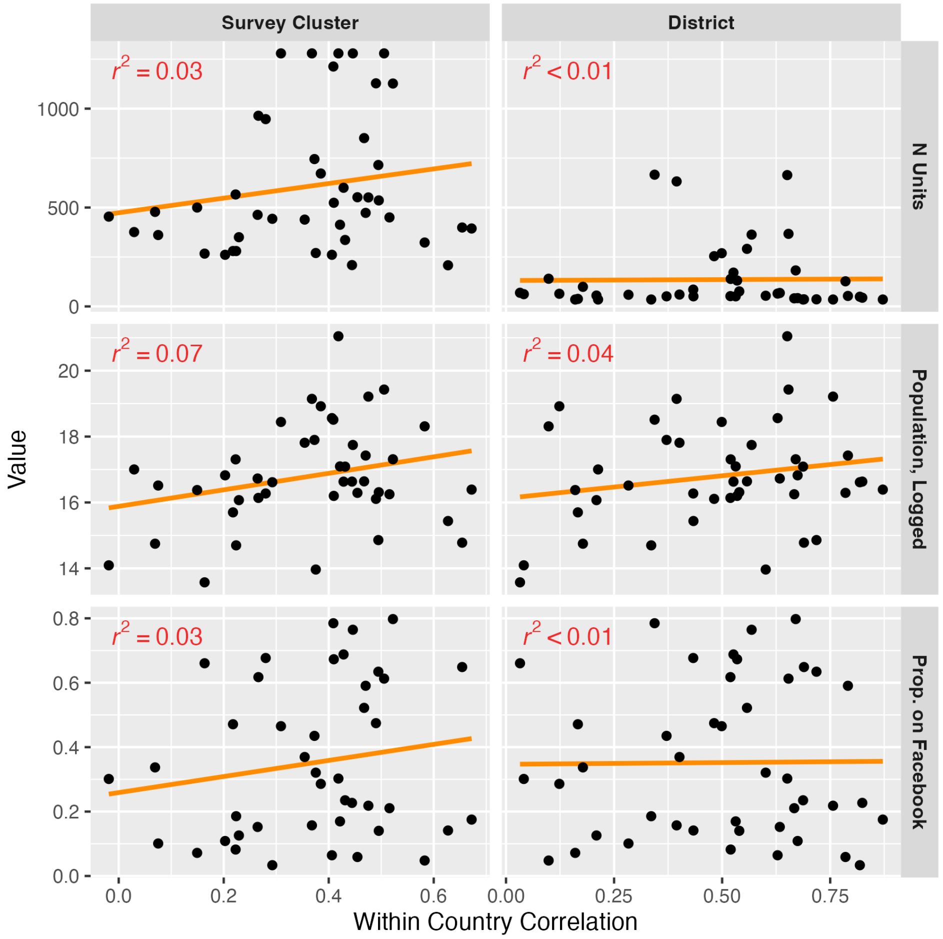

Figure S20: Scatterplot between (1) within-country correlation of the proportion with above high school education as measured by Facebook and DHS, and (2) country-level features

References

[1] Guanghua Chi, Han Fang, Sourav Chatterjee, and Joshua E. Blumenstock. Microestimates of wealth for all low- and middle-income countries. *Proceedings of the National Academy of Sciences*, 119(3):e2113658119, 2022.

[2] Neal Jean, Marshall Burke, Michael Xie, W Matthew Davis, David B Lobell, and Stefano Ermon. Combining satellite imagery and machine learning to predict poverty. *Science*, 353(6301):790–794, 2016.

[3] Christopher Yeh, Anthony Perez, Anne Driscoll, George Azzari, Zhongyi Tang, David Lobell, Stefano Ermon, and Marshall Burke. Using publicly available satellite imagery and deep learning to understand economic well-being in africa. *Nature Communications*, 11(1):2583, May 2020.
